# Controlling Iron Volatilization and Graphitization Through Pyrolysis in Benzoxazine Carbon Precursors

**DOI:** 10.1021/acsomega.6c04335

**Published:** 2026-06-30

**Authors:** Authors: Trey Schneider, Eric Williams, Anthony Nations, Penelope Jankoski, Tristan Clemons, Jeffrey Wiggins

**Affiliations:** School of Polymer Science and Engineering at the University of Southern Mississippi, Hattiesburg 39406, United States

## Abstract

In this work, two benzoxazine networks, BisA–An and BisA–Fu, were synthesized with and without 15 wt % ferrocene to investigate the influence of polymer network structure on iron volatilization and subsequent graphitization behavior. It is hypothesized that increased network rigidity, associated with higher cross-link density, would suppress iron volatilization during pyrolysis to 1000 °C, thereby increasing the availability of catalytically active iron at graphitization temperatures (>700 °C). Iron evolution during pyrolysis was monitored using simultaneous thermal analysis–mass spectrometry (STA–MS), which revealed that the network with the least cross-linking functionality exhibited approximately an order of magnitude greater iron volatilization compared to networks with higher cross-linking functionality (from 1.27 × 10^–10^ amps to 1.31 × 10^–11^ amps). X-ray diffraction (XRD) analysis showed corresponding differences in graphitic crystallite development, with a calculated crystallite thickness (Lc) of 3.44 nm in the sample with less cross-linking functionality and 4.31 nm observed in samples with higher cross-linking functionality. These results demonstrate that polymer network structure governs iron retention during pyrolysis and, consequently, influences the extent of catalytic graphitization.

## Graphical abstract

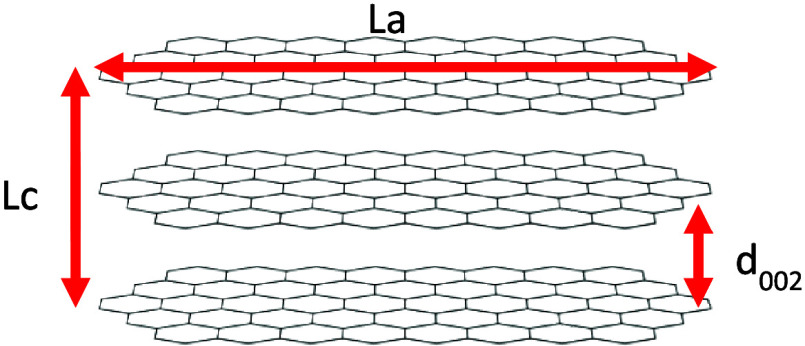

## Introduction

Carbon–carbon (C/C) composites consist of a carbon fiber reinforcement embedded within a carbonaceous matrix, most commonly derived from phenolic resins.^1^ Following curing, these composites are pyrolyzed at temperatures typically ranging from 800 to 3000 °C, during which heteroatoms are evolved and a carbon matrix is formed surrounding the carbon fiber reinforcement.^2^ Due to their exceptional thermal stability, low coefficient of thermal expansion, high specific strength, and resistance to thermal shock, C/C composites are widely used in extreme environments, including high-performance braking systems, rocket engine components, and thermal protection systems.^3^ Although carbon matrices can withstand temperatures approaching 3000 °C in inert atmospheres, their oxidation resistance in air is limited to approximately 500 °C. Consequently, ceramic coatings are commonly applied to C/C composites to form a passivating barrier that inhibits oxygen diffusion and mitigates oxidative degradation.^4^

Most thermosetting carbon precursors yield nongraphitizing “hard” carbons characterized by turbostratic morphology and significant open porosity.^5^ This structure facilitates oxygen diffusion and accelerates oxidative degradation. In contrast, graphitic carbons consist of stacked graphene layers (Figure 1) with reduced edge carbon density, resulting in improved oxidation resistance and oxidation onset temperatures that are typically 150–200 °C higher than those of amorphous carbons.^5^ The resistance of hard carbons to graphitization, even at temperatures exceeding 3000 °C, is generally attributed to the presence of rigid cross-linked networks that inhibit structural rearrangement into ordered graphitic crystallites.^6^ To overcome this limitation, metal-catalyzed graphitization has been widely investigated as a means of promoting graphitic ordering in hard carbons at reduced temperatures. Transition metals such as iron, cobalt, and nickel have been shown to promote the ordering of amorphous carbon through a dissolution–precipitation mechanism at elevated temperatures. In this process, carbon dissolves into the metal particles and subsequently precipitates as graphitic carbon on their surfaces, forming nanoscale ordered structures. This ordered carbon can then be measured by X-ray diffraction (XRD) picking out graphitic crystallite thickness (Lc), crystallite width (La), and interlayer spacing (d_002_).^7–9^ Prior studies have primarily focused on catalyst identity, particle size, processing temperature, and heating rate.^7,9–12^ While these parameters are known to influence graphitization efficiency, the role of the polymer chemistry, particularly the monomer and resulting network structure remains insufficiently understood.

**Figure 1:**
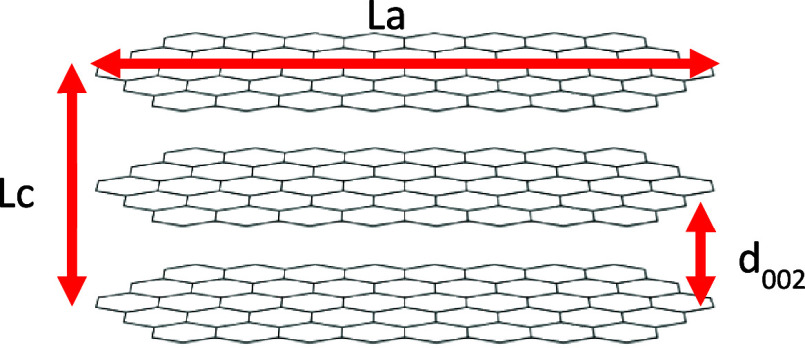
Depiction of a graphitic crystallite with dimensions Lc (crystallite thickness), La (crystallite width), and d_002_ (distance between graphene sheets).

Iron-catalyzed graphitization is described by a dissolution–precipitation mechanism, which becomes active at temperatures above ∼715 °C. In this process, amorphous carbon dissolves into iron particles and subsequently precipitates as graphitic carbon upon supersaturation. This precipitation is typically observed at temperatures approaching 750 °C, where graphitic layers begin to form on the surface of the iron particles.^11^ The iron catalyst can then continue to dissolve surrounding amorphous carbon and reprecipitate it as graphitic carbon, enabling sustained graphitic crystallite growth. Previous researchers have focused on factors such as catalyst source, catalyst type, particle size, graphitizing temperature, and pyrolysis ramp rate. Current trends show that iron, cobalt, and nickel are the most promising catalysts and that reducing metal catalyst size and increasing maximum temperature have the greatest influence on graphitic carbon formation.^9^

Currently, it remains unclear how the polymer matrix influences the efficiency and extent of metallocene-catalyzed graphitization. Talabi and co-workers investigated both commercial and laboratory-synthesized resoles containing varying amounts of ferrocene. In one study, they evaluated the graphitizing character by analyzing XRD patterns, where the area of the graphitic carbon peak was divided by the combined area of the graphitic and nongraphitic carbon peaks to determine the graphitic level (GL).^12^ Their results showed that increasing the formaldehyde-to-phenol ratio, which correspondingly increased the cross-link density of the polymer network, led to higher GL values.^12^ The maximum GL was observed at a formaldehyde-to-phenol ratio of 1.5, whereas the 2.0 ratio exhibited a comparatively lower GL. This reduction was attributed to excessive cross-link density within the network, which was proposed to hinder the ferrocene catalyzed graphitization.^12^ Similarly, Bitencourt and co-workers studied a Novolak and a coal-tar based resin with varying ferrocene content. In this study Bitencourt and co-workers observed that the ferrocene content, final pyrolysis temperature, time at final pyrolysis temperature, and heating rate can positively affect the extent of graphitization (using the same “Graphitization level” calculation).^10^ Additionally, the influence of cross-link density was also noted to affect the level of graphitization. With this observation, they concluded that increased cross-link density might inhibit the volatilization of iron through pyrolysis and that was the driving force for the changing graphitization level.^10^ Between these two papers, it is shown that cross-link density is another parameter that has not been directly studied and we hypothesize that cross-link density inhibits iron volatilization through pyrolysis and therefore controls the final concentration of iron and in turn, the extent of graphitization in a hard carbon sample.

In the present work, two similar benzoxazine precursor were chosen as they represent an attractive alternative to conventional phenolics due to their ability to cure without the evolution of volatile byproducts and their inherently high thermal stability (Td 5% or the temperature at which 5% degradation occurs) and char yield (remaining mass after pyrolysis), and a high degree of design flexibility.^13,14^ Benzoxazines are synthesized from phenols, formaldehyde, and amines, forming a characteristic oxazine ring (Figure 2 colored red). Bisphenol-A-based benzoxazines were synthesized using either aniline (Figure 2 left) or furfurylamine (Figure 2 right) as the amine components. Furfurylamine has been used previously to increase the cross-link density of benzoxazines, and this will act as the cross-link density control in the current body of work.^15,16^ Ferrocene, Figure 3, was incorporated as the iron-based graphitizing catalyst to evaluate how the polymer structure influences iron-catalyzed graphitization.

**Figure 2:**
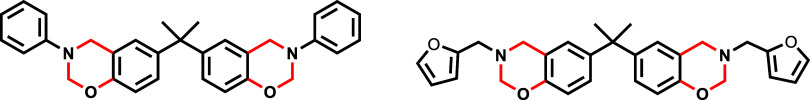
Bisphenol-A-Aniline (BisA-An) benzoxazine on the left and Bisphenol-A-Furfurylamine (BisA-Fu) benzoxazine on the right.

**Figure 3:**
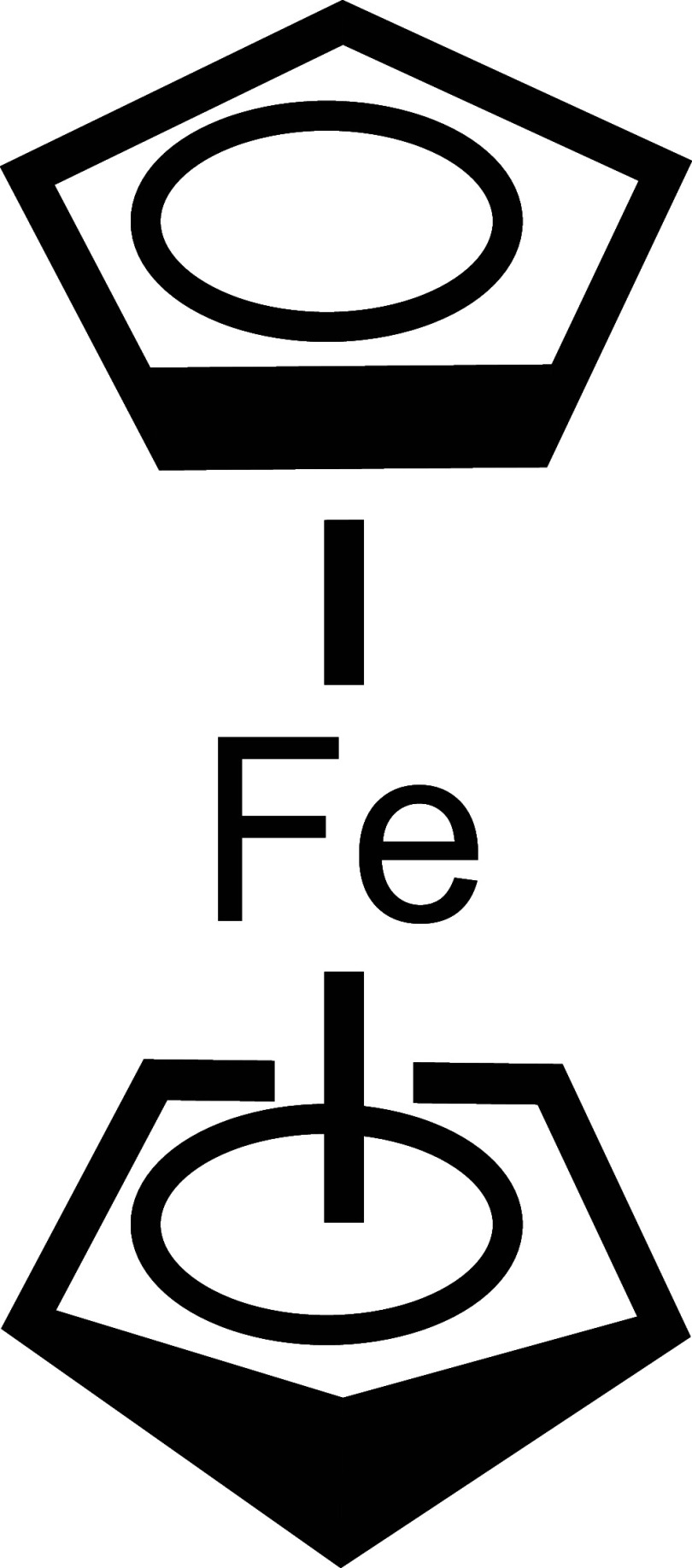
Ferrocene molecule utilized as the iron source in this study.

More specifically, bisphenol A-Aniline (BisA-An), bisphenol A-furfurylamine (BisA-Fu), and a 50/50 mix BisA-AnFu were each blended with 15 wt % ferrocene. Replacing aniline with furfurylamine introduces additional furan functionalities, thereby increasing the number of potential cross-linking sites within the polymer network. Across these three systems, the theoretical number of potential cross-linking sites increases from four in BisA-An, to an average of five in the 50/50 BisA-AnFu blend, and finally to six in BisA-Fu. We again hypothesize that cross-link density helps to control iron volatilization through pyrolysis and therefore controls the final concentration of iron and in turn, the extent of graphitization from a hard carbon source. In the present study, thermal analysis, mass spectrometry, X-ray diffraction, and electron microscopy were leveraged to elucidate the influence of the benzoxazine polymer structure on iron retention and the resulting extent of graphitic crystallite formation.

## Experimental Section

BisA-An and BisA-Fu benzoxazines were produced via a continuous reactor process utilizing a Coperion twin-screw extruder with an attached condenser assembly to capture water condensate. Bisphenol-A was solubilized in either aniline or furfurylamine and added as a single feed with a peristaltic pump. Additionally, formalin was fed with a separate peristaltic pump. The feed rates were calibrated to match the correct molar contribution of each component. All starting materials were added into the barrel sections where corotating screws imparted shear to adequately mix the reactants at elevated temperature. Barrel temperatures ranged from room temperature to 200 °C for BisA-An and from room temperature to 180 °C for BisA-Fu. At the end of the barrel, the benzoxazine product was discharged from the bottom of the die adapter into an aluminum pan, while steam exited through a crossover pipe mounted above. The final product was packaged and then stored in the freezer for further use. All process conditions and materials sources can be found in Tables 1,2,3, and[Table tbl4].

**Table 1:** Raw Materials Used for the Synthesis of BisA-An

reactants:	purity:	supplier/Lot #:
bisphenol-A	97%	alfa aesar lot: 10194727
aniline	99%	aldrich lot: MKCJ7129
formalin	37 wt % in water	ricca lot: 190936R

**Table 2:** Continuous High Shear Reactor Parameters for BisA-An Synthesis

zone 1 Feed: BPA-A mix	screw design: 17-USM.001	die adapter temp: no heat
zone 3 feed: formalin	screw speed: 200 rpm	chiller temp: 10 °C
zone 1 feed rate: 17.40g/min	zone 1 pump rate: 15.36 mL/min	zone 1 feed temp: 80 °C
zone 3 feed rate: 15.02g/min (10% excess)	zone 3 pump rate: 13.19 mL/min (10% excess)	zone 3 feed temp: RT

**Table 3:** Raw Materials Used for the Synthesis of BisA-Fu

reactants:	purity:	supplier/lot #:
bisphenol-A	97%	alfa aesar lot: 10194727
furfurylamine	98%	TCI lot: E436 K-LN
formalin	37 wt % in water	ricca lot: 2412C57

**Table 4:** Continuous High Shear Reactor Parameters for BisA-Fu Synthesis

zone 1 feed: BPA-Fu Mix	screw design: Fu BOX liquid feeds	die adapter temp: 100 °C
zone 3 feed: formalin	screw speed: 200 rpm	chiller temp: 10 °C
zone 1 feed rate: 21.67g/min	zone 1 pump rate: 19.37 mL/min	zone 1 feed temp: 60 °C
zone 3 feed rate: 18.37g/min (10% excess)	zone 3 pump rate: 15.66 mL/min (10% excess)	zone 3 feed temp: RT

Output rate and viscosity were monitored during each run, while proton NMR was performed after the run to confirm oxazine ring formation. NMRs of each monomer can be found in Supporting Information (Figures S1 and S2). Viscosity was measured with a Brookfield CAP 2000+ viscometer. BisA-An had a viscosity of 322 Poise at 80 °C with a rotation speed of 67 s^–1^ and BisA-Fu had a viscosity of 319 Poise at 85 °C with a rotation speed of 67 s^–1^.

Monomers were cured by ramping in a UF 55^PLUS^ Memmert oven from room temperature to 180 °C at a rate of 2 °C/min and held for 2 h, after which, the temperature was again ramped at 2 °C/min to 200 °C and held for 2 h. Thermogravimetric Analysis (TGA) was collected using a TA Q50. TGA samples were coarsely ground using a mortar and pestle and then 5 mg of sample was heated at a rate of 20 °C/min to 1000 °C under an inert nitrogen atmosphere. Differential scanning calorimetry (DSC) was conducted on a TA DSC2500. Two mg of monomer were heated at a rate of 5 °C/min from room temperature to 300 °C to observe cure exotherms. Dynamic mechanical analysis (DMA) was used to collect Tg values from cured networks with a thickness of 5 mm, width of 10 mm and length of 30 mm on a TA Instruments Q800 in tension mode. The DMA was heated from RT to 300 °C at 3 °C/min with a frequency of 1 Hz. Scanning electron microscope (SEM) powdered samples were adhered to SEM stubs using carbon tape and scanned as is. SEM Images were collected on a ZEISS SEM with an accelerating voltage of 20 kV and a working distance of 9.0 mm. X-ray diffraction (XRD) spectra were collected post pyrolysis on spinning carbon powder in an Anton Paar XRDynamic 500. Scans were taken between 2θ values of 15° and 89° with a step size of 0.02° and a time per step of 180.622s. A Netzsch simultaneous thermal analysis – mass spectrometer (STA-MS) or Netzsch STA 449 F3 Jupiter coupled with a Netzsch QMS 505 Rëolos was used to collect mass spectrometry of degradation gases between 50 and 1000 °C at a ramp rate of 20 °C/min under flowing helium and a scan range between 15 and 200 amu. Once a sample was loaded into the STA-MS, vacuum was pulled and backfilled with helium three times before starting the experiment with a constant flow of helium. Each scan was then analyzed through the Netzsch Proteus Thermal Analysis software. Transmission electron microscopy (TEM) was conducted on a JEOL JEM120i instrument at 120 kV through a range of magnification from 20k to 1.2 M. All samples were suspended in ethanol at 1 mg/mL and sonicated for a minimum of two hours to promote dispersion, subsequently 5 μL of sample was then dropped onto lacey carbon grids (purchased from Electron Microscopy Sciences in Hatfield, PA, USA) and air-dried overnight.

## Results and Discussion

### Differential Scanning Calorimetry

Differential scanning calorimetry (DSC) was used to evaluate differences in cure exotherms between neat and 15 wt % ferrocene-loaded benzoxazine samples (Figure 4). The corresponding polymerization exotherms and peak temperatures are summarized in Table 5. A slight increase in the initial polymerization onset is observed for all samples containing ferrocene (Figure 4). For the BisA-An system, the primary polymerization peak becomes slightly narrower with the addition of ferrocene. In the BisA-Fu system, the shoulder near 125 °C diminishes upon ferrocene incorporation. Additionally, Table 5 indicates that ferrocene on average increases the cure exotherm for the BisA-Fu system. For the BisA-AnFu mixture, the incorporation of ferrocene results in a transition from three distinct polymerization peaks to a single, broader feature, accompanied by a decrease in the overall polymerization exotherm.

**Figure 4:**
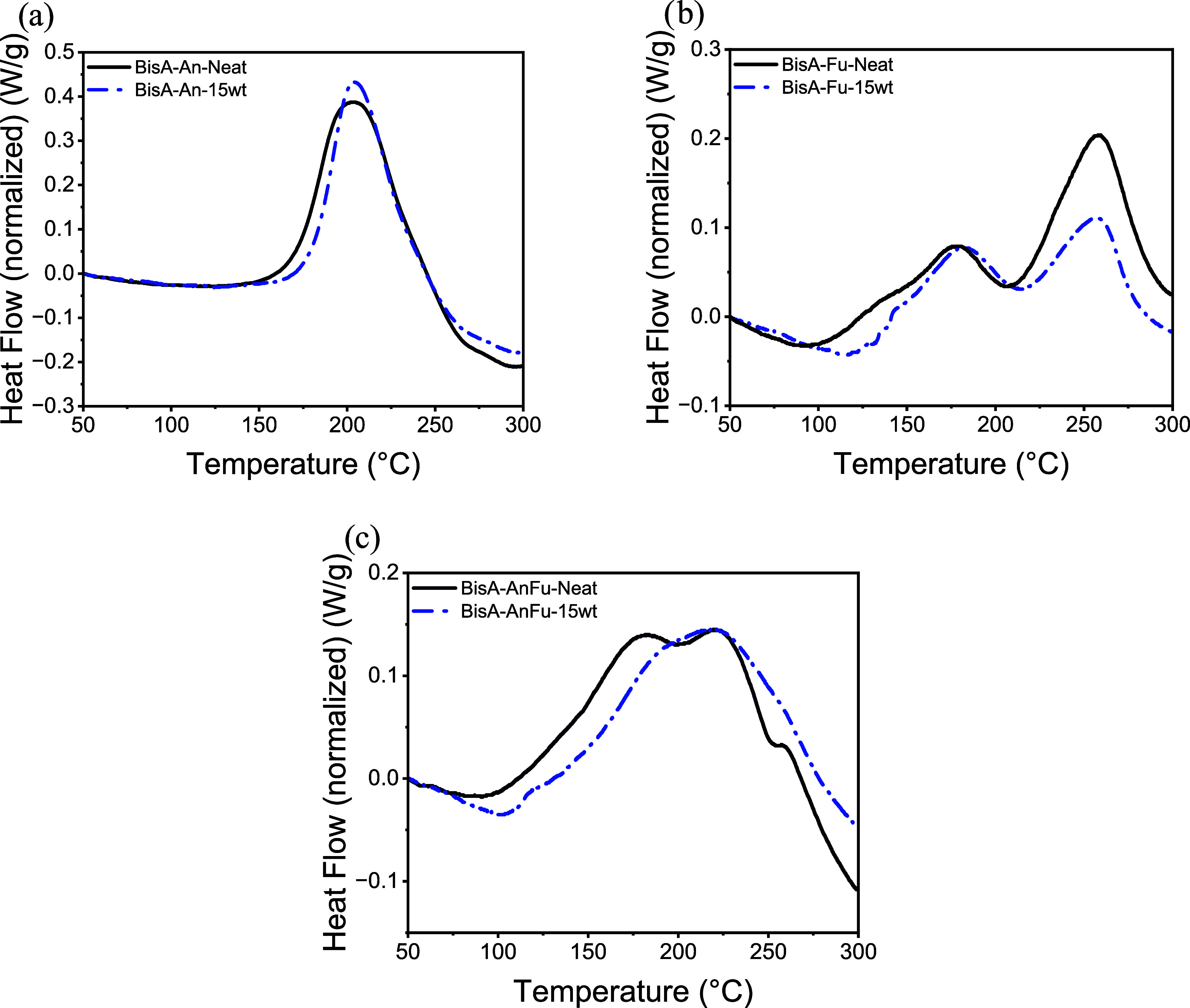
DSC of (a) Bis-A- Neat/BisA-An-15wt, (b) BisA-Fu-Neat/BisA-Fu-15wt, and (c) BisA-AnFu-Neat/BisA-AnFu-15wt from 50 to 300 °C at 5 °C/min.

**Table 5:** Table of DSC Polymerization Peaks and Exotherms for Each Material

	polymerization peak(s) (°C)	averaged exotherm (J/g)
BisA-An-Neat	214 ^± 0.70^	342.33 ^± 43.62^
BisA-An-15wt	219 ^± 1.01^	289.61 ^± 33.20^
BisA-AnFu-Neat	171 ^± 14.5^/221 ^± 1.73^/261 ^± 3.54^	289.34 ^± 11.49^
BisA-AnFu-15wt	218 ^± 4.62^	227.71 ^± 25.16^
BisA-Fu-Neat	191 ^± 0.35^/271 ^± 0.96^	123.98 ^± 14.00^
BisA-Fu-15wt	185 ^± 24.0^/262 ^± 18.71^	140.13 ^± 37.74^

Overall, the addition of ferrocene leads to a consistent decrease in polymerization exotherm for BisA-An and BisA-AnFu, while BisA-Fu saw an increased average cure exotherm. This behavior suggests that ferrocene may influence the polymerization process either by disrupting the benzoxazine curing mechanism or by effectively absorbing heat released from benzoxazine cure. However, due to the overlap of the 95% confidence intervals, no statistically significant effect can be conclusively established. While a detailed investigation is beyond the scope of this work, it is possible that ferrocene or its decomposition products interact with reactive intermediates during cure. For example, cyclopentadienyl species have been reported to act as radical scavengers, which could limit propagation by capping ring-opened benzoxazine structures.^17^

The BisA-An system exhibits a single polymerization exotherm, corresponding to the ring-opening polymerization of the benzoxazine ring. A simplified representation of the proposed curing mechanism is shown in Figure 5. Polymerization proceeds through a thermally accelerated mechanism where an impurity protonates the oxygen atom in the oxazine ring facilitating ring opening and the formation of tautomers. Electrophilic substitution can then occur at any activated carbon position on the aromatic ring. In systems containing furfurylamine, additional reactive sites are introduced through the furan ring, which can further participate in cross-linking reactions.^18–20^ The two systems incorporating furfurylamine exhibit additional polymerization peak(s), which are likely associated with secondary cross-linking reactions involving pendant furan groups. These reactions have been reported to occur at elevated temperatures, typically around 260 °C, and contribute to the more complex curing behavior observed in these systems.^19^

**Figure 5:**
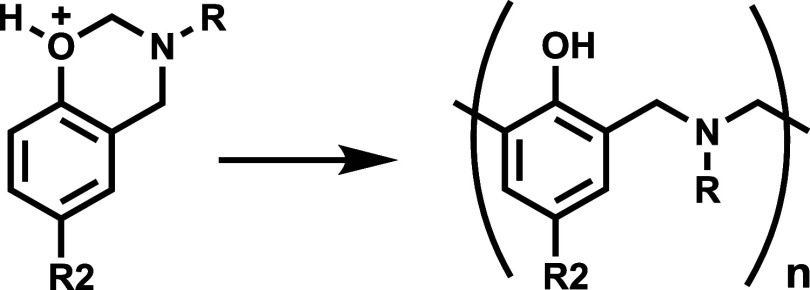
Simplified mechanism of benzoxazine polymerization.

### Dynamic Mechanical Analysis

Dynamic mechanical analysis (DMA) was conducted to evaluate changes in cross-link density with increasing furfurylamine content. Samples were cured according to the experimental procedure and subsequently heated from room temperature to 300 °C under tensile mode, with the glass transition temperature (Tg) determined from the peak of the tan δ curve (Figure 6). The measured Tg values and corresponding 95% confidence intervals are summarized in Table 6. The BisA-An-Neat sample exhibits the lowest Tg (172.20 °C), consistent with the expectation that aniline-based structures contribute fewer additional cross-linking sites, resulting in a less rigid network. The tan δ response for this sample shows a primary Tg peak near 172 °C, followed by a secondary feature above 250 °C, which is attributed to the onset of thermal degradation and/or additional thermally induced cross-linking. In contrast, BisA-AnFu-Neat and BisA-Fu-Neat display higher Tg values of 245.25 and 253.89 °C, respectively, with overlapping 95% confidence intervals. Both systems exhibit broad tan δ transitions, and the corresponding storage modulus versus temperature curves (Figures S3–S5) do not display a well-defined rubbery plateau up to 300 °C, due to additional cure and degradation. Regardless, cross-link density was estimated according to the classical theory of rubbery elasticity.^21^ The rubbery modulus and temperature T were taken at Tg + 30 with calculated results in Table 6. It should be noted that this approach is most appropriate for lightly cross-linked rubbers exhibiting a stable rubbery plateau above *T*_g_.^21^ Although these materials do not fully satisfy those assumptions, it still provides a useful relative comparison between networks. The calculated values indicate that increasing furfurylamine content leads to a substantial increase in cross-link density (Table 6). The calculated cross-link density for BisA-An appears reasonable; however, the furfurylamine-containing networks undergo additional cross-linking and thermal degradation above *T*_g_, resulting in an increase in storage modulus above Tg, and consequently, an artificially elevated calculated cross-link density. Despite this limitation, the overall trend in calculated cross-link density remains consistent with the measured *T*_g_values, suggesting that BisA-An-Neat possesses the lowest cross-link density, while BisA-AnFu-Neat and BisA-Fu-Neat exhibit progressively higher cross-link densities.

**Figure 6:**
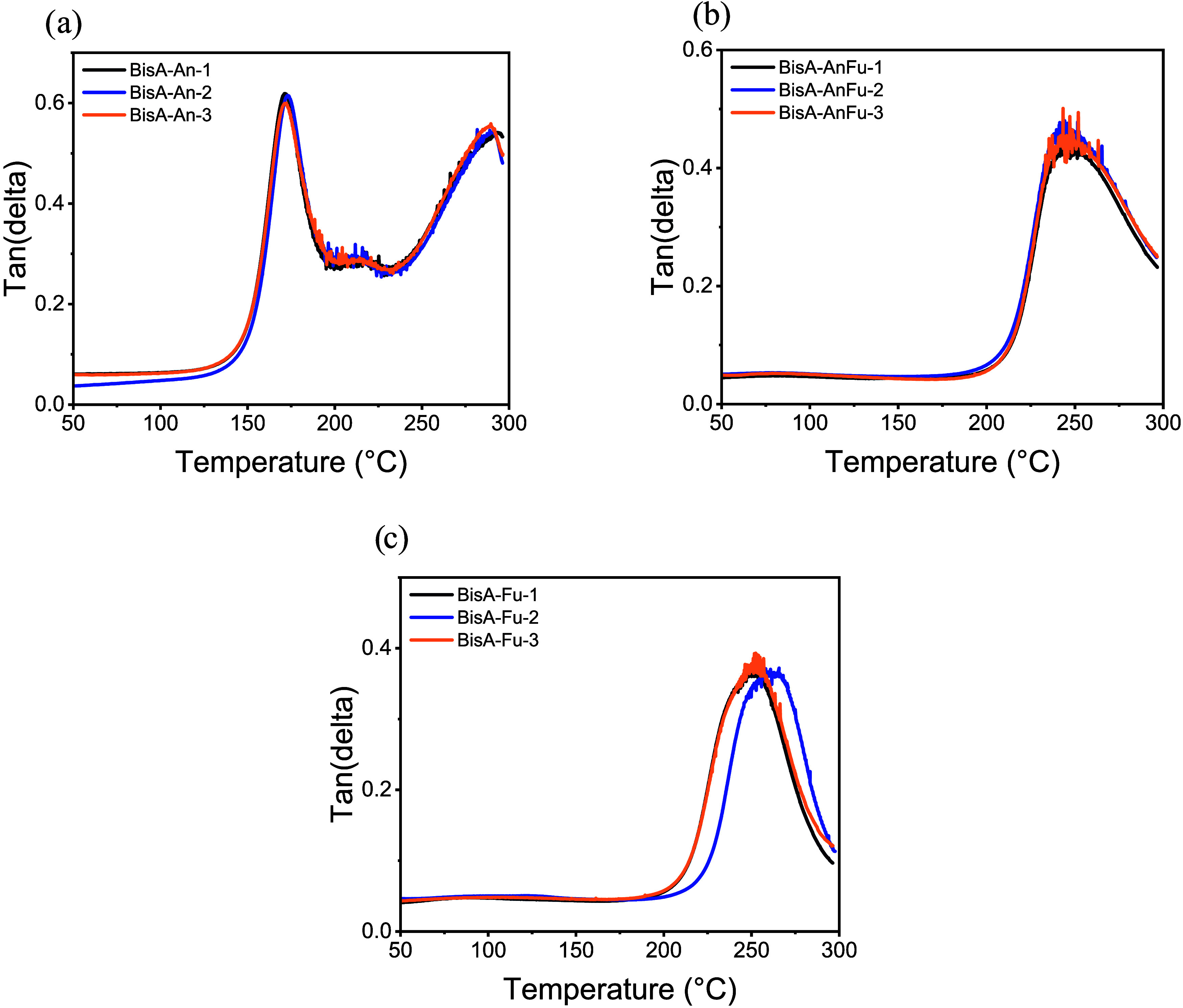
DMAs of each neat sample collected in triplicate (a) BisA-An-Neat, (b) BisA-AnFu-Neat, and (c) BisA-Fu-Neat.

**Table 6:** Calculated Tg Based on the Peak of Tan Delta with Error Bars and Estimated Crosslink Densities for Each System

	Tg (°C)	cross-link density (mol/m3)
BisA-An-Neat	172.20 ^± 2.05^	3,423
BisA-AnFu-Neat	245.25 ^± 6.67^	1,086,164
BisA-Fu-Neat	253.89 ^± 9.23^	5,337,823

### Thermogravimetric Analysis

Thermogravimetric analysis (TGA) was conducted to assess the differences in nonisothermal degradation profiles according to monomer structure and ferrocene incorporation. Previous work by Ran et.al. reported mass loss between 200 and 300 °C in aniline-based systems, attributed to the degradation and subsequent volatilization of aniline.^22^ In the present study, a similar trend is observed, where initial degradation occurs between 250 and 350 °C across all systems (Figure 8). Considering the results from their study, it is likely that any uncross linked furans or anilines are the first to cleave from the BisA networks. A depiction of these first degradation events is shown in Figure 7 as blue dashed lines.

**Figure 7:**
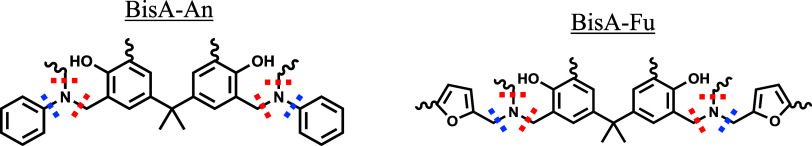
Anticipated degradation points in each polymer system with the low temperature degradation (>250 °C) shown by a blue line and higher temperature degradation (>350) shown with a red line.

The initial mass loss region provides the basis for determining the 5% degradation temperature (Td_5%_) for each system. The Td_5%_ values and corresponding char yields are summarized in Table 7. All ferrocene-containing samples exhibit significantly lower Td_5%_ values compared to their neat counterparts, which is attributed to the volatilization of ferrocene species from the surface of the polymer matrix. As the temperature exceeds Tg, increased segmental mobility may facilitate the diffusion of ferrocene to the surface, resulting in earlier volatilization and mass loss. As seen by moulijn and co-workers these metal particles can become mobile even at low temperatures.^23^ Systems containing furfurylamine are expected to form more highly cross-linked networks, which may restrict mass transport and delay the evolution of volatile species to higher temperatures. The primary degradation step for all samples occurs between 350 and 600 °C and is attributed to main chain scission involving nitrogen-containing linkages (Figure 7, red dashed lines). Prior work by Hemvichian et al. suggests that larger amines favor C–N bond cleavage, leading to the formation of Schiff base structures, whereas smaller amines more readily undergo C–C bond scission.^24^ Based on this, aniline and furfurylamine are expected to behave as bulkier amines, with degradation proceeding predominantly through C–N bond cleavage. For systems containing furan functionality, an additional degradation event is observed between 600 and 800 °C. This is likely associated with the continued evolution of aromatic species such as substituted phenols, benzenes, and higher molecular weight compounds including biphenyl, naphthalene, and benzofuran.^25^ which were likely held into the network due to the additional cross-linking functionality added by the furfurylamine groups. These species may be retained to higher temperatures due to the increased cross-link density and aromatic character introduced by the furfurylamine groups, resulting in delayed volatilization.

**Table 7:** Table of 5% Degradation Temperature (Td 5%) and Char Yield of Each Material

N_2_ atmosphere	Td 5% (°C)	char yield (%)
BisA-An-Neat	322.3 ^± 6.5^	23.16 ^± 0.73^
BisA-An-15wt	242.7 ^± 31.9^	23.22 ^± 7.80^
BisA-AnFu-Neat	337.0 ^± 4.1^	39.99 ^± 2.52^
BisA-AnFu-15wt	269.0 ^± 15.4^	44.26 ^± 4.33^
BisA-Fu-Neat	331.1 ^± 9.0^	46.13 ^± 1.71^
BisA-Fu-15wt	291.8 ^± 4.2^	51.05 ^± 3.36^

Considering the char yields at 1000 °C, the results indicate an overall increase in char yield with both ferrocene incorporation and increasing furfurylamine content. The BisA-An systems exhibit the lowest char yield (∼23%), with no statistically significant difference between the neat and ferrocene-loaded samples. Based on ferrocene composition, iron (56 amu) accounts for approximately 4.5 wt % of the total 15 wt % ferrocene (186 amu) added to each sample, while the remaining ∼10.5 wt % corresponds to the cyclopentadienyl ligands, which are expected to volatilize during degradation. From this, it would be anticipated that ferrocene-containing samples would exhibit a reduction in char yield of approximately 10.5 wt % from the ligands and a 4.5% boost in char yield from the iron. This would result in a net 6% loss in char yield relative to their neat counterparts. However, this expected decrease is not observed. Instead, the char yields remain constant or increase slightly with ferrocene incorporation. For the BisA-An system, the comparable char yields between neat and ferrocene-loaded samples suggest that either iron is volatilizing during pyrolysis or that iron is promoting the retention of additional carbon within the char, offsetting the expected mass loss. In the BisA-AnFu and BisA-Fu systems, a modest increase in average char yield is observed with ferrocene addition. While these differences are not statistically significant based on the 95% confidence intervals, the trend is consistent with the weight contribution from retained iron species. These observations suggest that ferrocene influences degradation through multiple competing mechanisms. Previous work by Karakozova et al. proposed that cyclopentadienyl species contain abstractable hydrogen atoms capable of stabilizing degrading polymer chains through radical capping.^17^ Such interactions could promote char formation by limiting chain scission and enhancing carbon retention. Conversely, these same species may also interfere with curing reactions by scavenging reactive intermediates, potentially reducing cross-link density and negatively impacting char yield. Additionally, Iron has been used as a catalyst for carbon–carbon bond formation, meaning that it is likely catalyzing carbon coupling within the degrading network leading to additional carbon retention and increased char yields.^26,27^ Overall, the curing and degradation behavior of benzoxazine systems in the presence of ferrocene appears to be convoluted and warrants further investigation (Figure 8).

**Figure 8:**
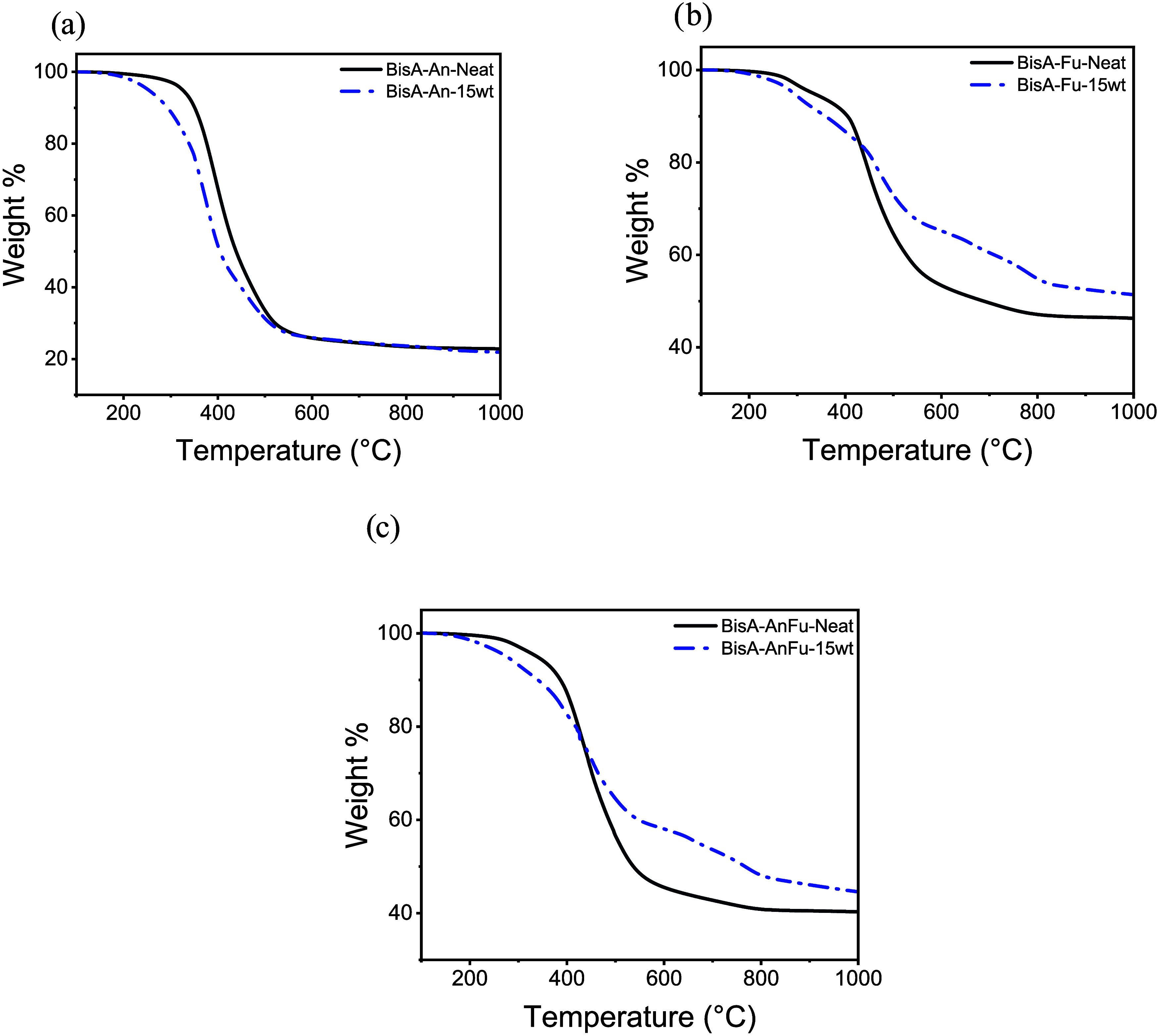
TGA of (a) Bis-A- Neat/BisA-An-15wt, (b) BisA-Fu-Neat/BisA-Fu-15wt, and (c) BisA-AnFu-Neat/BisA-AnFu-15wt from 50 to 1000 °C at 10 °C/min.

### Simultaneous Thermal Analysis – Mass Spectrometry

A simultaneous thermal analyzer coupled with mass spectrometry (STA–MS) was used to evaluate the evolution of iron-containing species during pyrolysis. This technique operates similarly to thermogravimetric analysis (TGA), where the sample is heated under a controlled atmosphere, in this case helium, while continuously monitoring mass loss. The evolved gases are transferred to the mass spectrometer, where ion current is recorded as a function of mass-to-charge ratio (*m*/*z*), corresponding to specific atomic mass units (amu), as shown in Figure 9.

**Figure 9:**
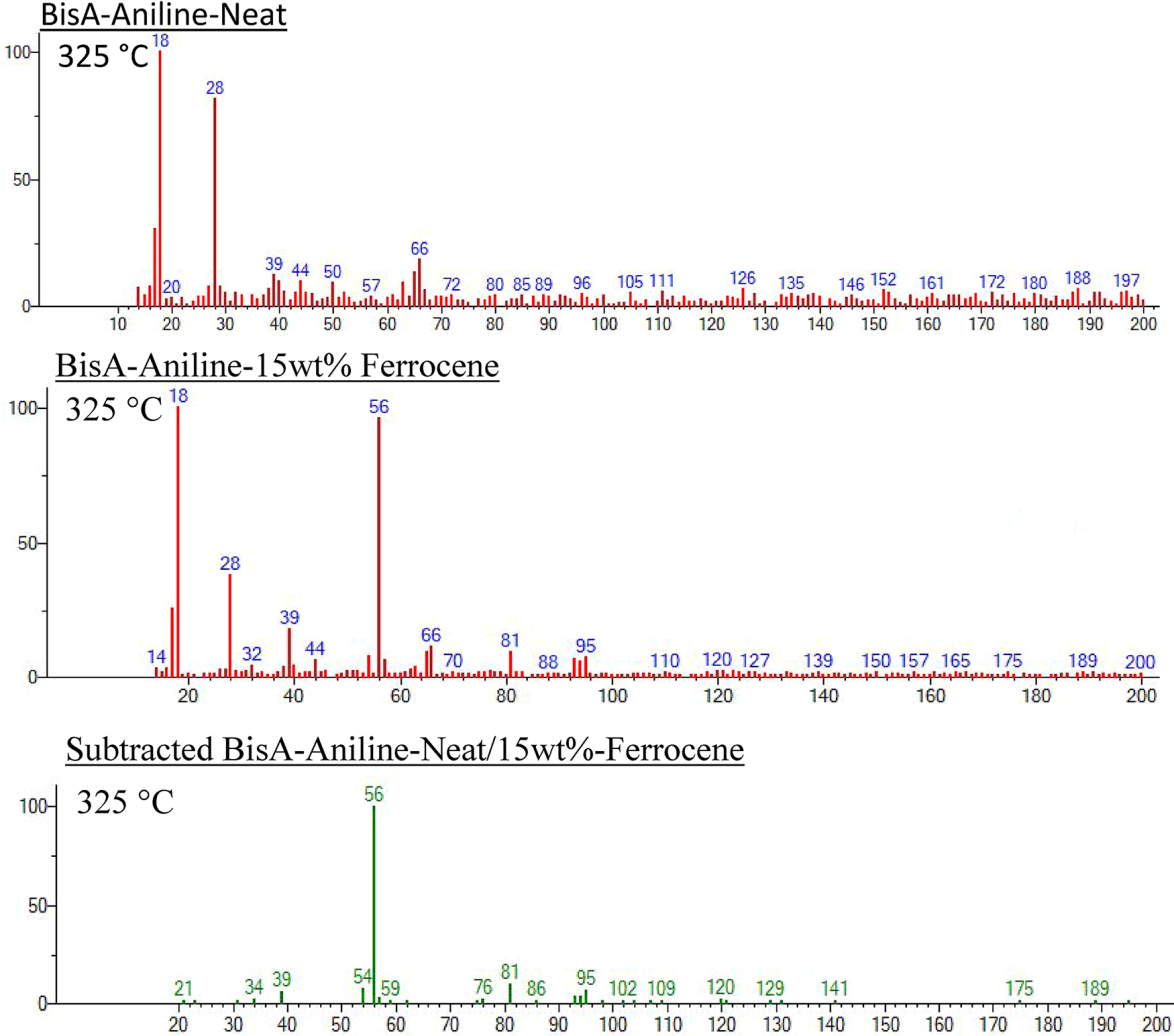
STA-MS scan for BisA-Aniline-Neat at 325 °C (TOP), BisA-Aniline-15 wt %-Ferrocene at 325 °C (middle), and subtracted spectra (bottom).

Figure 9 presents MS spectra for BisA-An-Neat and BisA-An containing 15 wt % ferrocene at 325 °C. For the neat sample, two primary signals are observed at 18 and 28 amu, corresponding to water and carbon monoxide, respectively. The ferrocene-containing sample exhibits these same features, along with an additional signal at 56 amu. Subtraction of the neat spectrum from the ferrocene-loaded spectrum highlights this difference, with the 56 amu peak emerging as the dominant distinguishing feature. This signal is attributed to iron derived from ferrocene decomposition. While assignment of a single *m*/*z* signal in evolved gas analysis can be influenced by fragmentation overlap, the absence of a corresponding 56 amu signal in the neat benzoxazine sample strongly supports its association with iron generated during ferrocene decomposition. The presence of this peak indicates that iron is released in a volatile form during pyrolysis, suggesting that not all iron remains within the carbon matrix. This volatilization is expected to influence the extent of catalytic graphitization in ferrocene-containing systems. It is hypothesized that as the temperature exceeds Tg, increased segmental mobility facilitates diffusion of volatile iron-containing species through the polymer network and toward the particle surface, resulting in iron volatilization between approximately 200 and 400 °C. low temperature metal particle mobility has also been observed in the past by Moulijn and co-workers supporting this idea.^23^ All samples were cured to a maximum temperature of 200 °C prior to this experiment, which coincides with the onset of iron evolution observed by STA-MS. DSC experiments further show that all samples continue to exhibit an exothermic response up to 300 °C, indicating that additional cure and/or degradation reactions are occurring during this temperature range. The increase in chain mobility associated with temperatures above Tg likely permits the volatilization of iron, and enabling its detection in the STA-MS. As curing progresses, the network structure becomes increasingly rigid and Tg shifts to higher temperatures, reducing segmental motion and restricting mass transport within the material. Furfurylamine-containing benzoxazines are expected to form more highly cross-linked networks, as supported by the calculated cross-link density values. The increased cross-link density reduces free volume and decreases the diffusion coefficient as the size and connectivity of transient diffusion pathways within the polymer matrix decreases.^28^ In addition, the more rigid network structure suppresses large-scale chain rearrangements that would otherwise facilitate migration of iron particles. Together, these effects hinder diffusion of volatile iron species, and thereby promote iron retention above 400 °C.

Figure 10 shows the evolution of the 56 amu signal as a function of temperature up to 1000 °C for all ferrocene-loaded samples. The BisA-An-15 wt % sample exhibits a significantly higher integrated intensity (approximately an order of magnitude, 1.27 × 10^–10^ amps) than either the BisA-AnFu-15 wt % (2.15 × 10^–11^ amps) or BisA-Fu-15 wt % (1.31 × 10^–11^ amps) systems. This suggests a greater extent of iron volatilization in the BisA-An system, while the furfurylamine-containing systems retain more iron during pyrolysis. Based on this observation, it is reasonable to expect that BisA-AnFu-15 wt % and BisA-Fu-15 wt % will exhibit similar extents of graphitization, as the amount of iron retained to catalyze graphitic ordering is comparable. Interestingly, both furfurylamine-containing systems exhibit similar iron retention despite differences in calculated cross-link density. This suggests that there is an upper limit of required cross-link density to inhibit the volatilization of iron, and additional increases in cross-link density provide diminishing improvements in the suppression of iron volatilization.

**Figure 10:**
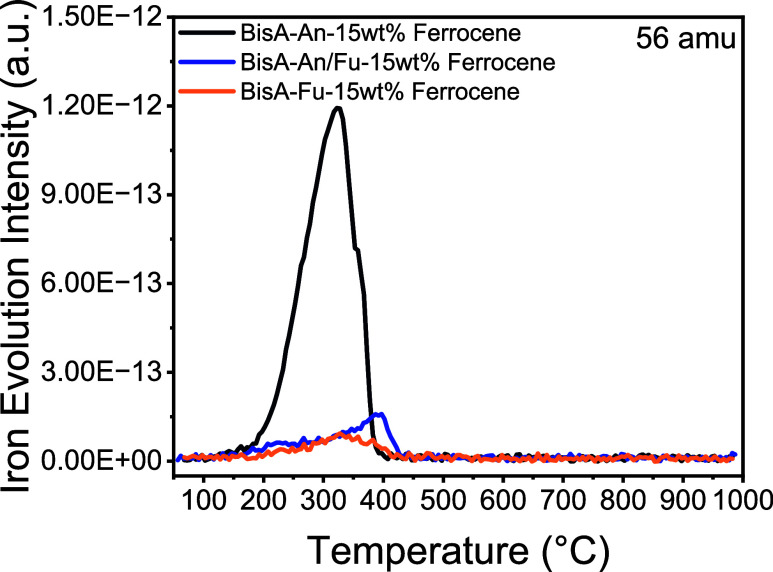
Ion current of iron evolution through pyrolysis of each ferrocene loaded sample.

### X-ray Diffraction

Current understanding, supported by in situ XRD studies, suggests that iron catalyzes the graphitization of amorphous carbon through a dissolution–precipitation mechanism.^29^ Upon heating, ferrocene decomposes to form metallic iron at approximately 400 °C, which can subsequently react with carbon to form iron carbide near 715 °C.^30^ At higher temperatures (∼750 °C), this carbide becomes metastable and decomposes into γ-iron and graphitic carbon. The regenerated γ-iron can then continue to dissolve surrounding amorphous carbon, reforming iron carbide and repeating the cycle, ultimately promoting graphitic crystallite growth.^11^

X-ray diffraction (XRD) was used to quantify the resulting carbon structure through calculation of the graphitic crystallite thickness (Lc), lateral size (La), and interlayer spacing (d_002_) (see Figure 1). The (002) reflection near 26° was used to determine both Lc and d_002_, while the (100)/(101) region near 43° is typically used for La. Unfortunately, due to overlap between the La peak and iron carbide peaks, La could not be calculated in this study. The crystallite thickness (Lc) was calculated using the Scherrer equation. where *K* is a shape factor (0.93), λ is the X-ray wavelength (0.15406 nm), β is the full width at half-maximum (fwhm) of the (002) peak, and θ is the Bragg angle.^31^ The interlayer spacing (d_002_) was calculated using Bragg’s law based on the same (002) peak position.^32^ The calculated values for all samples are summarized in Table 8.

**Table 8:** Calculated Lc and d002 Values from all the XRD Spectrum Shown in Figure 11

	700 °C (Lc/d_002_)		1000 °C (Lc/d_002_)	
BisA-An-Neat	1.08 nm	3.70 Å	1.03 nm	3.79 Å
BisA-An-15wt	0.98 nm	3.70 Å	3.44 nm	3.44 Å
BisA-AnFu-Neat	1.10 nm	3.80 Å	1.07 nm	3.74 Å
BisA-AnFu-15wt	1.03 nm	3.62 Å	4.30 nm	3.41 Å
BisA-Fu-neat	1.07 nm	3.66 Å	1.13 nm	3.81 Å
BisA-Fu-15wt	1.02 nm	3.48 Å	4.31 nm	3.42 Å

All XRD patterns collected between 15° and 90° for samples pyrolyzed at 700 °C (blue) and 1000 °C (red) are shown in Figure 11 a–f. The neat benzoxazine-derived carbons (Figure 11 a–c) exhibit broad (002) reflections characteristic of amorphous carbon, with calculated Lc values of approximately 1 nm.^33^ This confirms that, in the absence of a catalyst, these materials remain largely disordered and do not undergo significant graphitization at these temperatures. In contrast, the ferrocene-containing samples (Figure 11 d–f) show clear structural evolution. After pyrolysis at 700 °C, the (002) peak remains broad, indicating limited graphitic ordering; however, additional reflections appear above 30°, corresponding to iron and iron carbide phases. Peaks observed near 44°, 65°, and 83° are assigned to the (110), (200), and (211) planes of body-centered cubic (BCC) iron, while peaks near 36.72°, 39.72°, 42.82°, 43.64, 57.97°, and 68.76° are consistent with iron carbide phases.^34^ Overlap between these reflections and the (100)/(101) region (∼43°) prevents reliable determination of La without peak deconvolution. Notably, the BisA-An-15 wt % sample shows minimal evidence of iron carbide formation after pyrolysis at 700 °C, which is consistent with earlier STA–MS results indicating greater iron volatilization in this system. Upon pyrolysis to 1000 °C, all ferrocene-containing samples exhibit significant sharpening of the (002) peak, accompanied by increases in calculated Lc values (Table 8), indicating enhanced graphitic crystallite growth. Corresponding iron and carbide reflections also become more pronounced, although the BisA-An-15 wt % sample shows only a modest increase in iron peak intensity, further supporting reduced iron retention.

**Figure 11:**
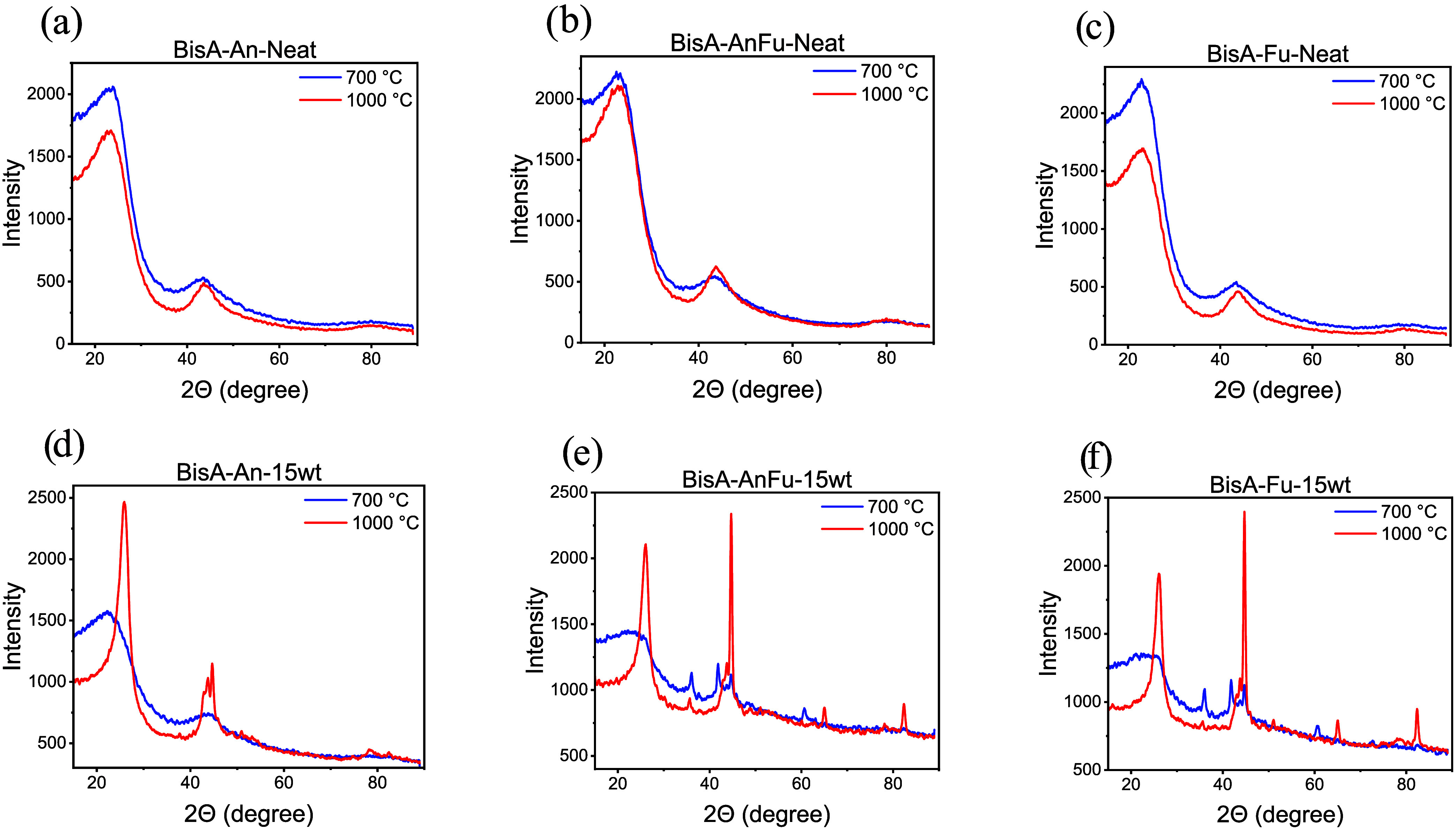
Graphs of all XRD samples showing all the ’Neat’ samples pyrolyzed at 700 and 1000 °C on the top row (a-c) and all the 15 wt % loaded samples pyrolyzed at 700 and 1000 °C on the bottom row (d-f).

The calculated d_002_ values provide additional insight into crystallite packing. Values greater than ∼3.44 Å indicate turbostratic or poorly ordered carbon, whereas values approaching 3.35 Å correspond to well-ordered graphite.^33^ The lowest d_002_ value observed in this study is 3.42 Å for the BisA-Fu-15 wt % sample pyrolyzed to 1000 °C. While Lc increases with temperature, indicating growth of graphitic domains, the relatively large d_002_ values suggest that these crystallites remain imperfectly stacked. It is also important to note that these values represent averages over the entire sample, which are likely to contain a mixture of graphitic and amorphous carbon.

To further illustrate these trends, Lc values were plotted as a function of material for both 700 and 1000 °C pyrolysis conditions (Figure 12). All neat samples maintain Lc values near 1 nm regardless of temperature, confirming the absence of significant graphitization. In contrast, ferrocene-containing samples show substantial increases in Lc at 1000 °C. As anticipated based on STA–MS results, the BisA-An-15 wt % sample exhibits a lower Lc value, consistent with greater iron loss during pyrolysis. Additionally, the similar extent of iron evolution observed for the BisA-AnFu-15 wt % and BisA-Fu-15 wt % systems correspond to comparable Lc values. As discussed previously, the similar iron volatilization behavior of the furfurylamine-containing systems suggests that once sufficient network rigidity is achieved, further increases in cross-link density may have diminishing effects on restricting iron transport during pyrolysis. Both systems are therefore likely sufficiently cross-linked, producing similar iron retention and resulting graphitization values. These results suggest that the extent of graphitic crystallite development is governed primarily by the availability of iron retained during pyrolysis, rather than the initial benzoxazine structure. While polymer chemistry influences degradation behavior and iron retention, the final degree of graphitization appears to be controlled by the effective concentration of catalytic iron present at elevated temperatures.

**Figure 12:**
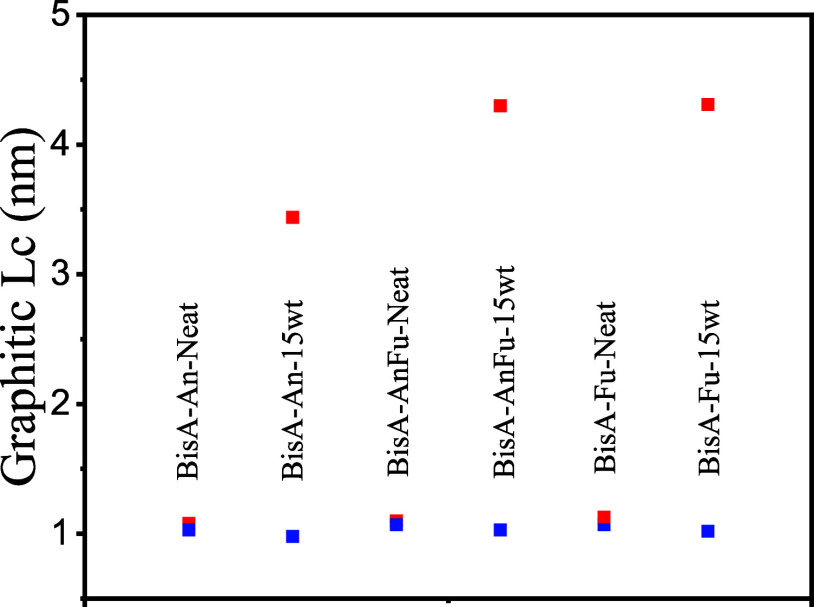
Plotted Lc of each sample with the 700 °C pyrolysis samples in blue and 1000 °C pyrolysis samples in red.

### Scanning Electron Microscopy (SEM) and Transmission Electron Microscopy (TEM)

To better understand the dispersion of iron species within the polymer matrix, scanning electron microscopy (SEM) and transmission electron microscopy (TEM) were employed. The ferrocene additive initially consisted of particles approximately 100–200 μm in size prior to blending. Upon incorporation into the molten benzoxazine monomers, the ferrocene appeared to dissolve into the matrix, with no visible phase separation after removal of magnetic stirring. Based on this observation, the resulting iron dispersion length scale after processing was not immediately apparent. SEM imaging (Figure 13) did not reveal any distinct iron agglomerates down to a resolution of ∼200 nm, suggesting that any iron-containing domains are below this size scale. SEM was therefore primarily used to assess particle size following the grinding process, which produced particles below 10 μm in diameter. Additional SEM images are provided in the Supporting Information (Figures S6–S9).

**Figure 13:**
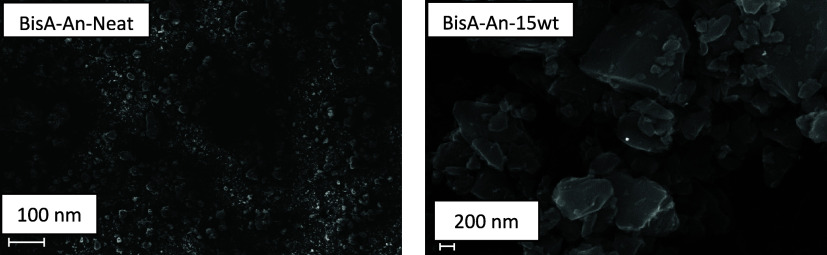
SEM images of BisA-An-Neat (Left) and BisA-An-15wt (Right).

TEM was subsequently used to directly probe the nanoscale dispersion of iron and to assess the development of graphitic carbon. Representative images of BisA-An-Neat and BisA-An-15 wt % samples are shown in Figure 14 (50 nm scale), with additional images provided in Supporting Information (Figures S10–S17). In the ferrocene-containing sample, small dark inclusions are observed that are absent in the neat material. These features are attributed to iron particles and are estimated to be on the order of ∼10 nm or smaller. This indicates that the micron-scale ferrocene particles are effectively dispersed to the nanoscale during processing, likely through dissolution and subsequent redistribution within the monomer matrix rather than simple mechanical size reduction. Higher magnification TEM images (20 nm scale, Figure 15) further confirm the presence of sub-10 nm iron-containing inclusions in the ferrocene-loaded samples. At 700 °C, no clear evidence of graphitic ordering is observed in either the neat or ferrocene-containing systems, which is consistent with XRD results and the expectation that catalytic graphitization requires higher temperatures.

**Figure 14:**
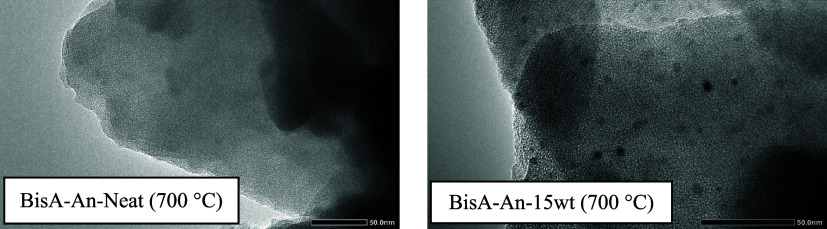
50 nm TEM images of BisA-An-Neat (left) and BisA-An-15wt (right) pyrolyzed to 700 °C.

**Figure 15:**
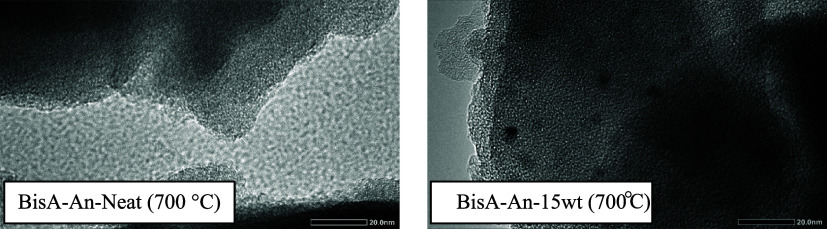
Twenty nm TEM images of BisA-An-Neat (left) and BisA-An-15wt (right) pyrolyzed to 700 °C.

After pyrolysis at 1000 °C, clear differences emerge (Figure 16). The neat benzoxazine-derived carbon remains largely featureless, with no indication of graphitic ordering. In contrast, the ferrocene-containing sample exhibits regions of increased contrast and texture, suggesting the formation of nanoscale graphitic domains. At higher magnification (20 nm scale, Figure 17), the iron-containing sample reveals distinct core–shell structures, consisting of dark iron-rich particles surrounded by lighter, ordered carbon layers. Due to the relatively small catalyst particle size, graphitic ordering appears to proceed through a static growth mechanism. Goldie and co-workers reported that iron particles below approximately 15.9 nm preferentially form encapsulating graphitic shells rather than migrate through the carbon matrix to produce carbon nanotubes.^35^ Image analysis of these features (Figure 18) using ImageJ indicates that the graphitic shell thickness ranges from approximately 4.3 to 4.5 nm. This value is larger than the average crystallite thickness (Lc) obtained from XRD, which is expected since XRD provides a bulk-average measurement that includes both amorphous and poorly ordered regions. In contrast, TEM selectively captures well-developed local structures, such as graphitic shells surrounding iron particles. These observations support the conclusion that localized catalytic graphitization occurs at iron particle surfaces, resulting in the formation of nanoscale graphitic shells during pyrolysis at 1000 °C.

**Figure 16:**
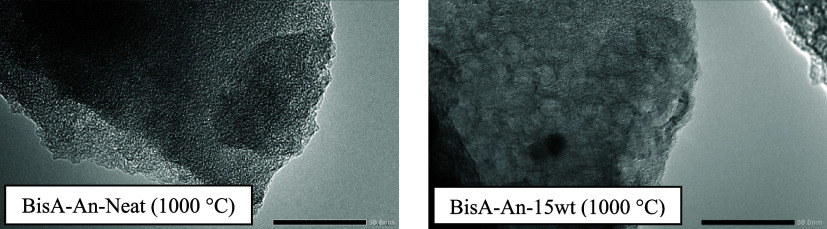
50 nm TEM images of BisA-An-Neat (left) and BisA-An-15wt (right) pyrolyzed to 1000 °C.

**Figure 17:**
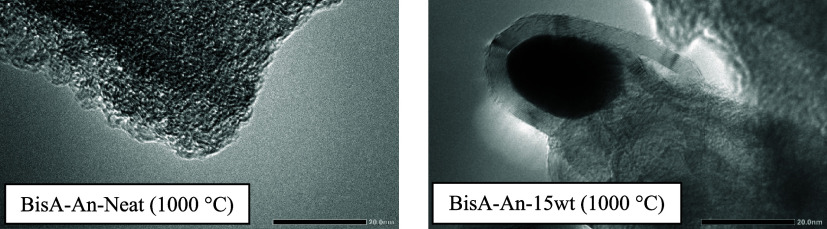
Twenty nm TEM images of BisA-An-Neat (left) and BisA-An-15wt (right) pyrolyzed to 1000 °C.

**Figure 18:**
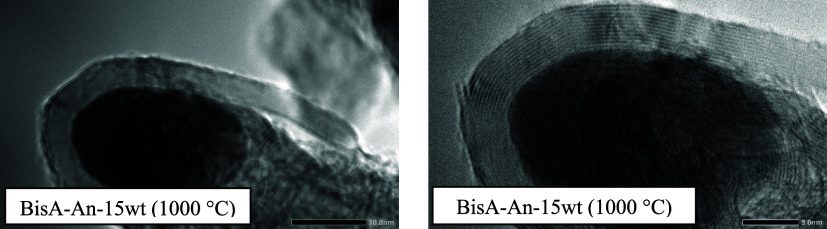
TEM images of BisA-An-15wt at 10 nm scale (left) and BisA-An-15wt at 5 nm scale (right) pyrolyzed to 1000 °C.

## Conclusion

In this study, the influence of benzoxazine network structure on iron-catalyzed graphitization was investigated through pyrolysis at 700 and 1000 °C. STA–MS results confirmed that iron-containing species volatilize during pyrolysis, with significantly greater loss observed in polymers systems with less cross-linking functionality. Based on these findings, it is proposed that cross-link density plays a key role in limiting iron mobility and volatilization, thereby controlling the amount of iron retained at elevated temperatures. X-ray diffraction further demonstrated that increased iron retention leads to enhanced graphitic crystallite development, as evidenced by higher Lc values. Together, these results indicate that the extent of graphitization in these systems is governed primarily by the availability of catalytic iron during pyrolysis, rather than the initial polymer chemistry alone. During these experiments additional avenues of study became apparent, such as, the true curing mechanism of benzoxazines in the presence of ferrocene and the true degradation mechanisms of benzoxazines in the presence of ferrocene.

## Supplementary Material



## Notes

NMRs of monomers used in this study; graphs providing Storage and Loss moduli for all cured samples without ferrocene; additional SEMs of all samples with and without ferrocene after 700 °C pyrolysis; and additional TEMs of all samples with and without ferrocene after 700 and 1000 °C pyrolysis (PDF)
