# Supplementary material for: Controlling Iron Volatilization and Graphitization Through Pyrolysis in Benzoxazine Carbon Precursors
Source: ACS Omega. 2026 Jun 30;11(27):40745–57. doi: 10.1021/acsomega.6c04335 (PMC13382830; doi:10.1021/acsomega.6c04335)
Supplement: Supplementary file 1 [file ao6c04335_si_001.pdf]

Controlling Iron Volatilization and Graphitization through Pyrolysis in Benzoxazine Carbon Precursors. – Supporting Information

Authors: Trey Schneider [a], Eric Williams [a], Anthony Nations [a], Penelope Jankoski [a], Tristan Clemons [a], Jeffrey Wiggins* [a]

[a] School of Polymer Science and Engineering at The University of Southern Mississippi, 39406, USA.

Corresponding Author at the School of Polymer Science and Engineering at The University of Southern Mississippi, 118 College Dr. #5050, Hattiesburg, Mississippi 39406, USA. E-mail address: Jeffrey.Wiggins@usm.edu

Abstract

In this work, two benzoxazine networks, BisA–An and BisA–Fu, were synthesized with and without 15 wt% ferrocene to investigate the influence of polymer network structure on iron volatilization and subsequent graphitization behavior. It is hypothesized that increased network rigidity, associated with higher crosslink density, would suppress iron volatilization during pyrolysis to 1000 °C, thereby increasing the availability of catalytically active iron at graphitization temperatures (>700 °C). Iron evolution during pyrolysis was monitored using simultaneous thermal analysis–mass spectrometry (STA–MS), which revealed that the network with the least crosslinking functionality exhibited approximately an order of magnitude greater iron volatilization compared to networks with higher crosslinking functionality (from $1.27x10^{-10}$ amps to $1.31x10^{-11}$ amps). X-ray diffraction (XRD) analysis showed corresponding differences in graphitic crystallite development, with a calculated crystallite thickness (Lc) of 3.44 nm in the sample with less crosslinking functionality and 4.31 nm observed in samples with higher crosslinking functionality. These results demonstrate that polymer network structure governs iron retention during pyrolysis and, consequently, influences the extent of catalytic graphitization.

Contents

The following data is found in the Supplementary Information document:

- NMRs of the starting monomers. (S1 and S2)
- Storage modulus and loss modulus results for each neat network. (S3 – S5)
- SEMs of both neat and ferrocene filled furfurylamine networks pyrolyzed to 700 °C. (S6 – S9)
- TEMs of all networks neat and filled pyrolyzed at 700 °C and 1000 °C. (S10 – S17)

NMR of as synthesized monomers

The following NMRs correspond to the monomers utilized in this work

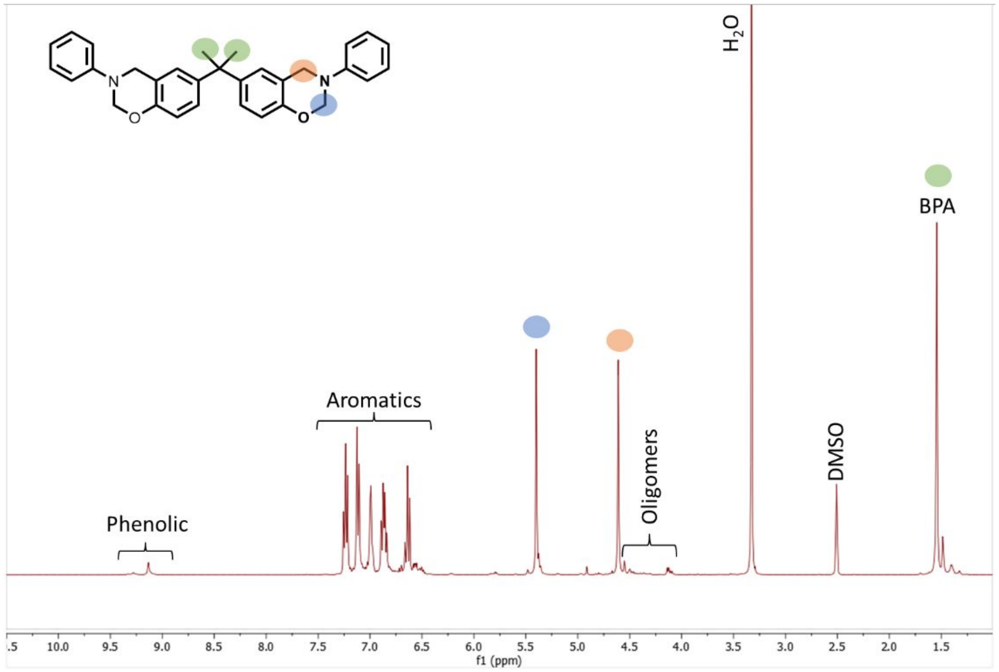

Figure S1: NMR of BisA-An as synthesized monomer (10 mg sample in 500 µl of d-DMSO).

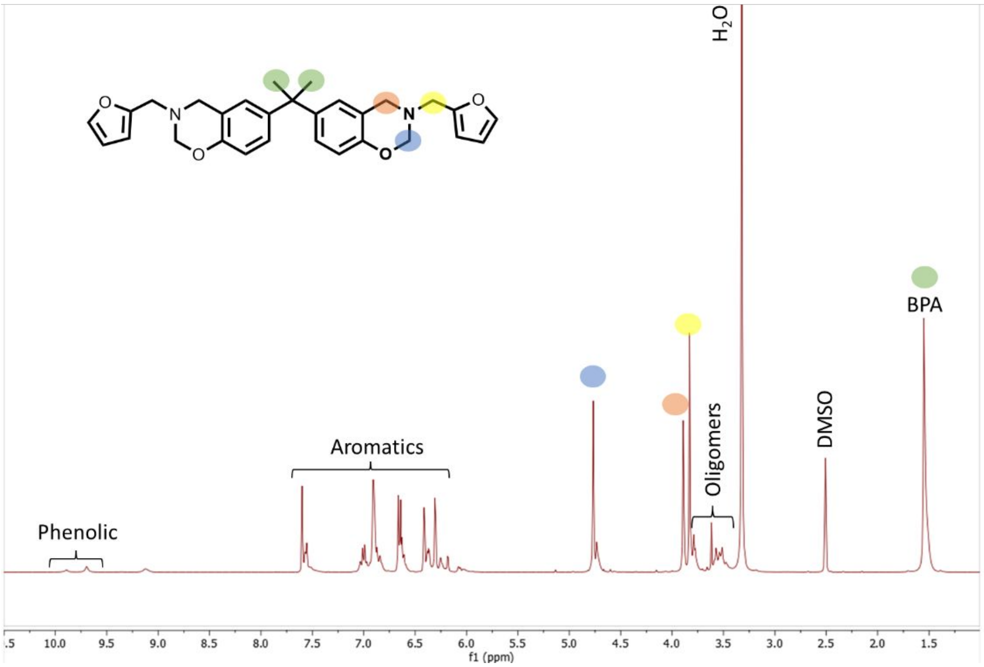

Figure S2: NMR of BisA-Fu as synthesized monomer (10 mg sample in 500 µl of d-DMSO).

DMA

The following graphs provide Storage and Loss moduli for all cured neat samples

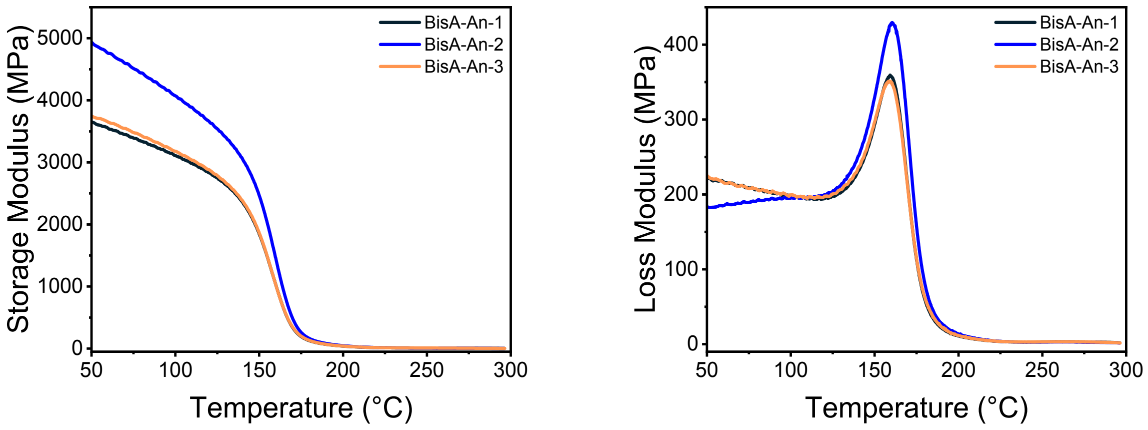

Figure S3: Storage modulus and Loss modulus for BisA-An collected on DMA.

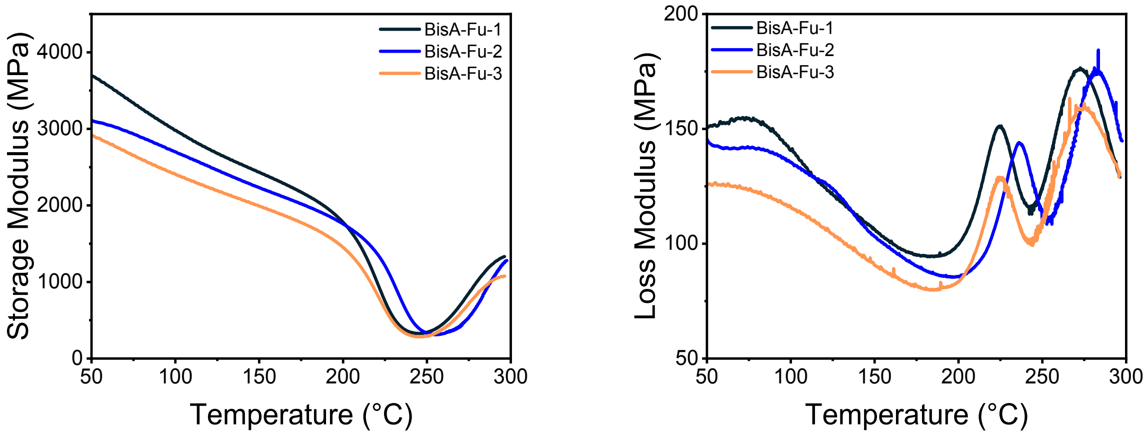

Figure S4: Storage modulus and Loss modulus for BisA-Fu collected on DMA.

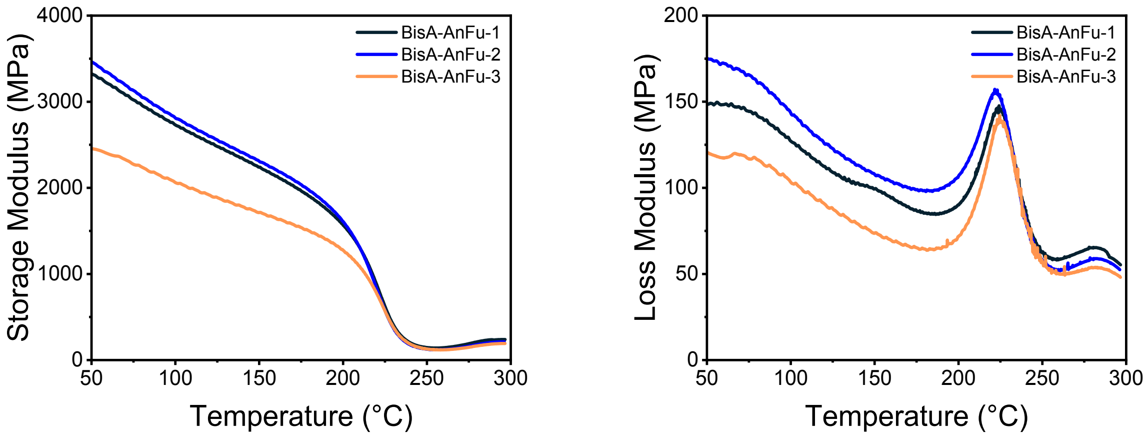

Figure S5: Storage modulus and Loss modulus for BisA-AnFu collected on DMA.

SEM Images

Additional SEM images of all samples with and without ferrocene

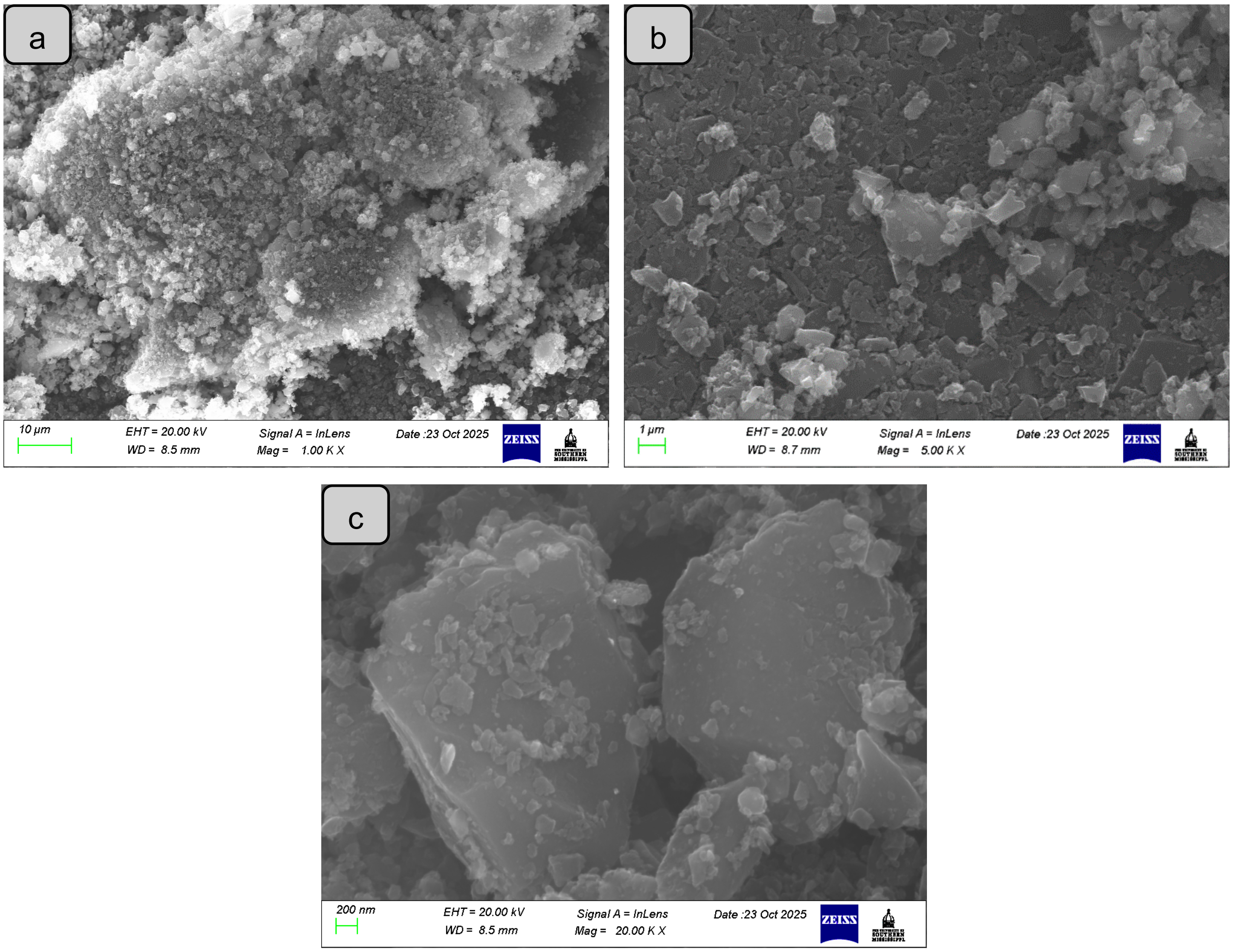

Figure S6: SEM images of BisA-Fu-15wt 700 °C from 10 µm (a) to 200 nm (c).

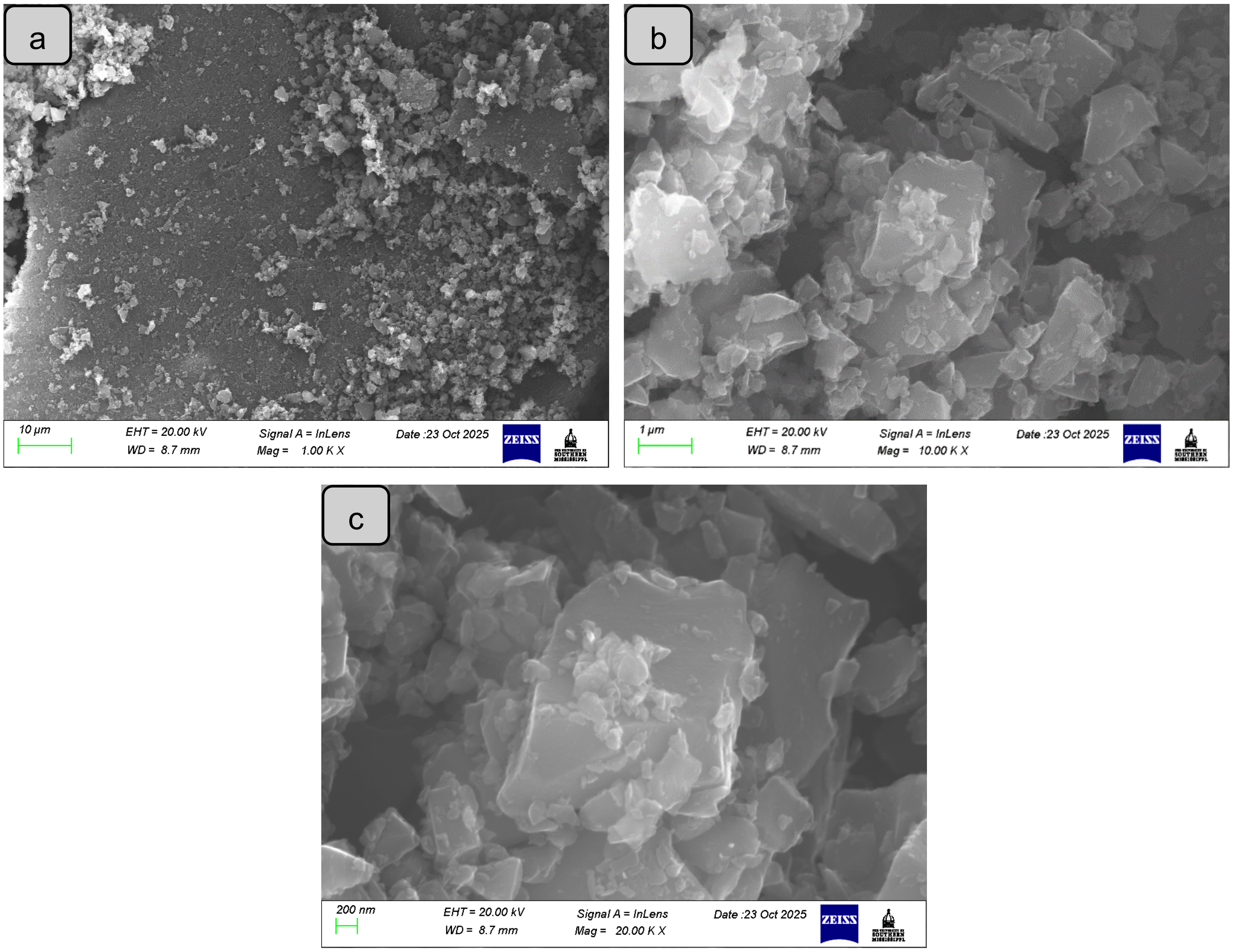

Figure S7: SEM images of BisA-Fu-Neat 700 °C from 10 µm (a) to 200 nm (c).

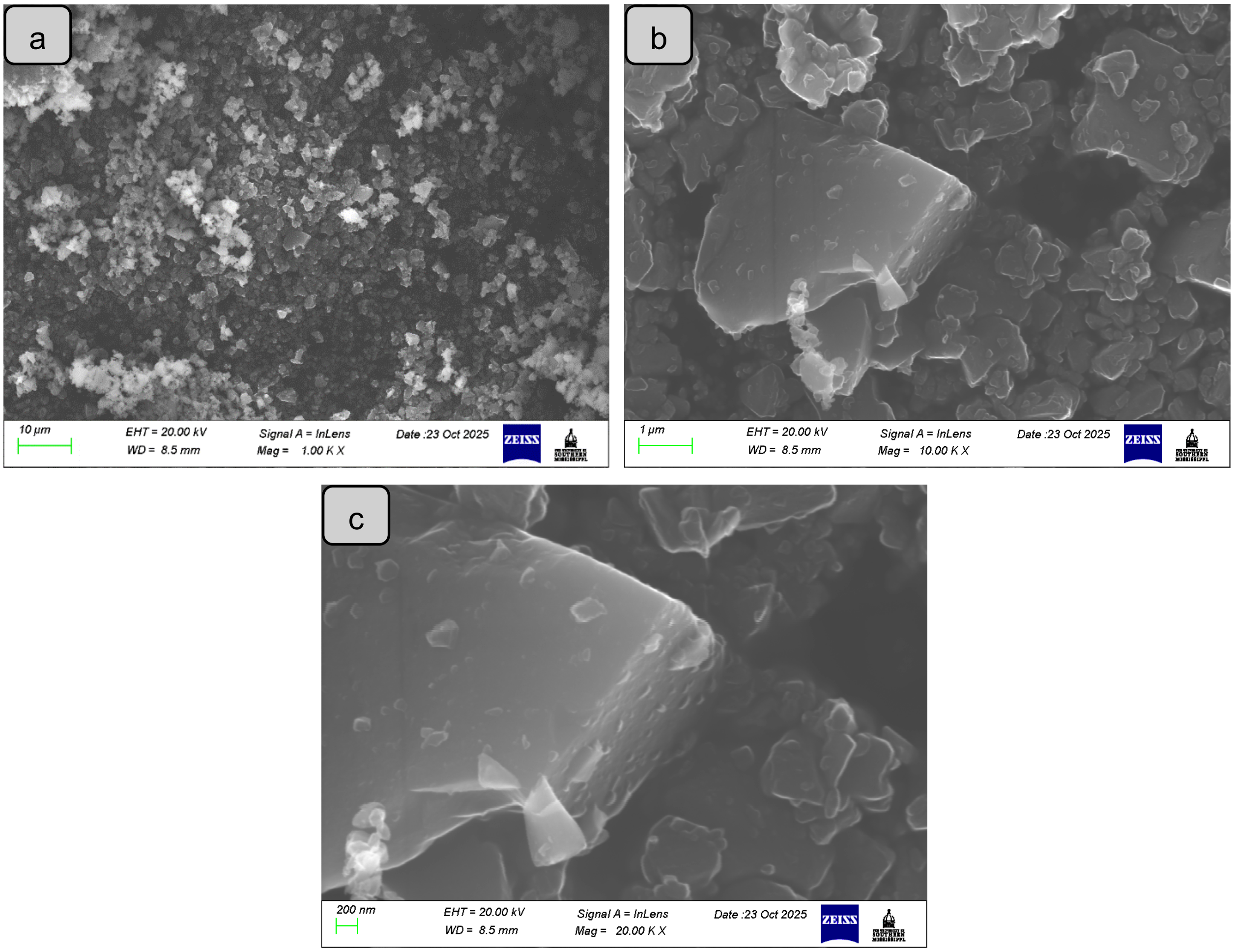

Figure S8: SEM images of BisA-AnFu-Neat 700 °C from 10 µm (a) to 200 nm (c).

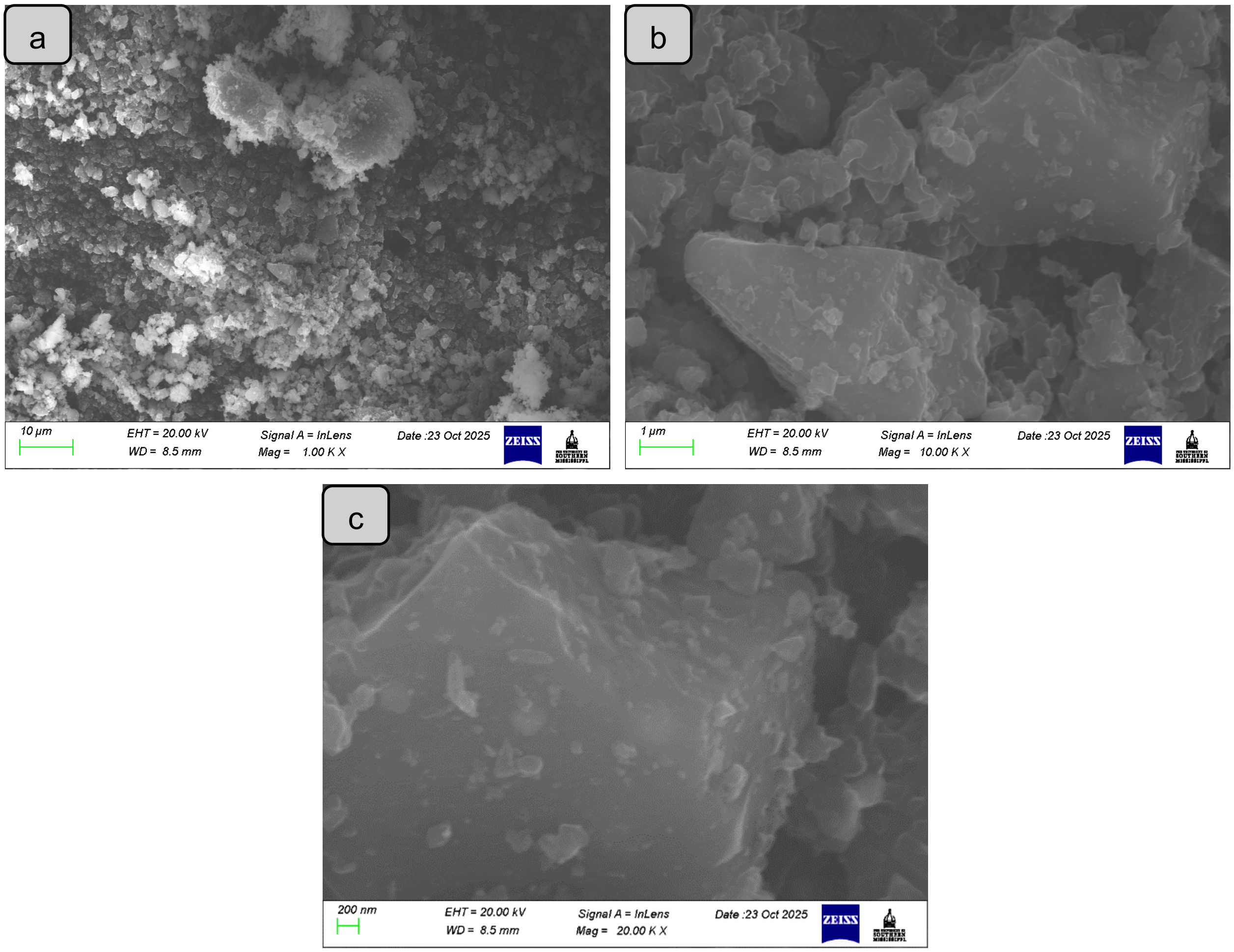

Figure S9: SEM images of BisA-AnFu-15wt 700 °C from 10 µm (a) to 200 nm (c).

TEM Images

Additional TEMs of all samples with and without ferrocene

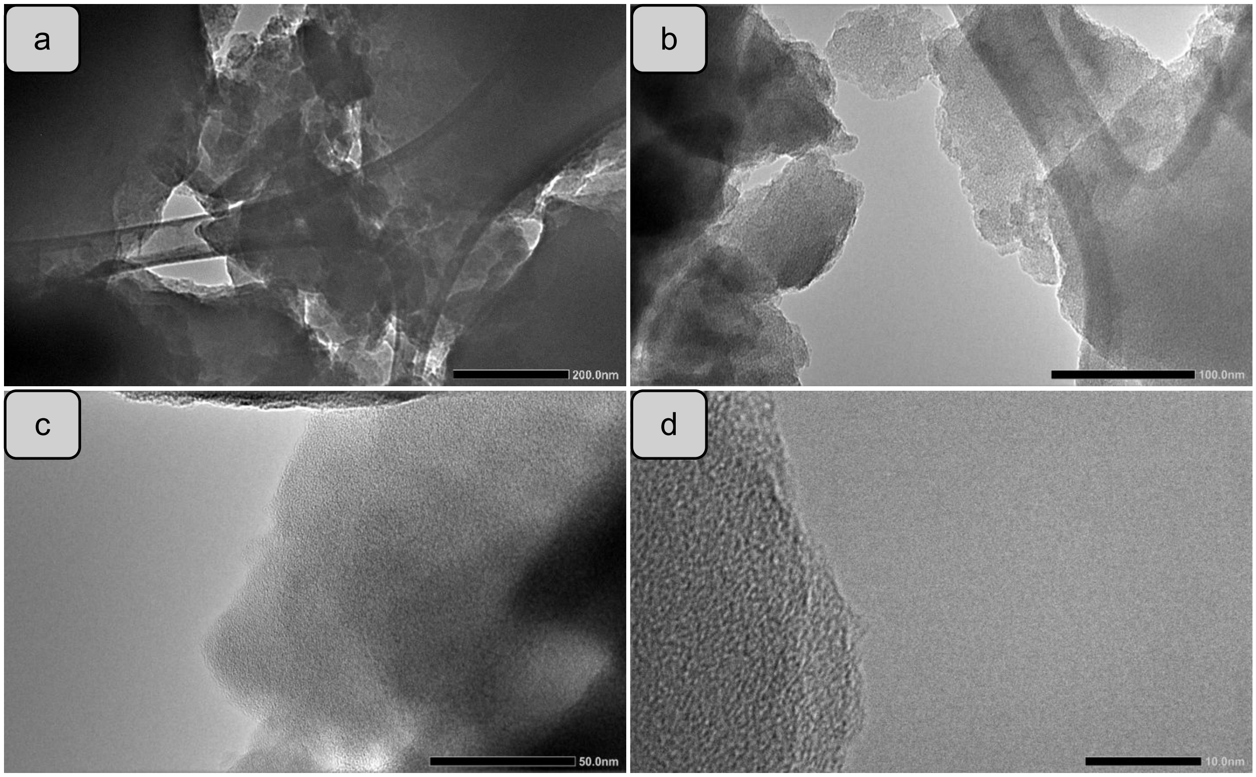

Figure S10: TEM images of BisA-Fu-Neat 700 °C from 200 nm (a) to 10 nm (d).

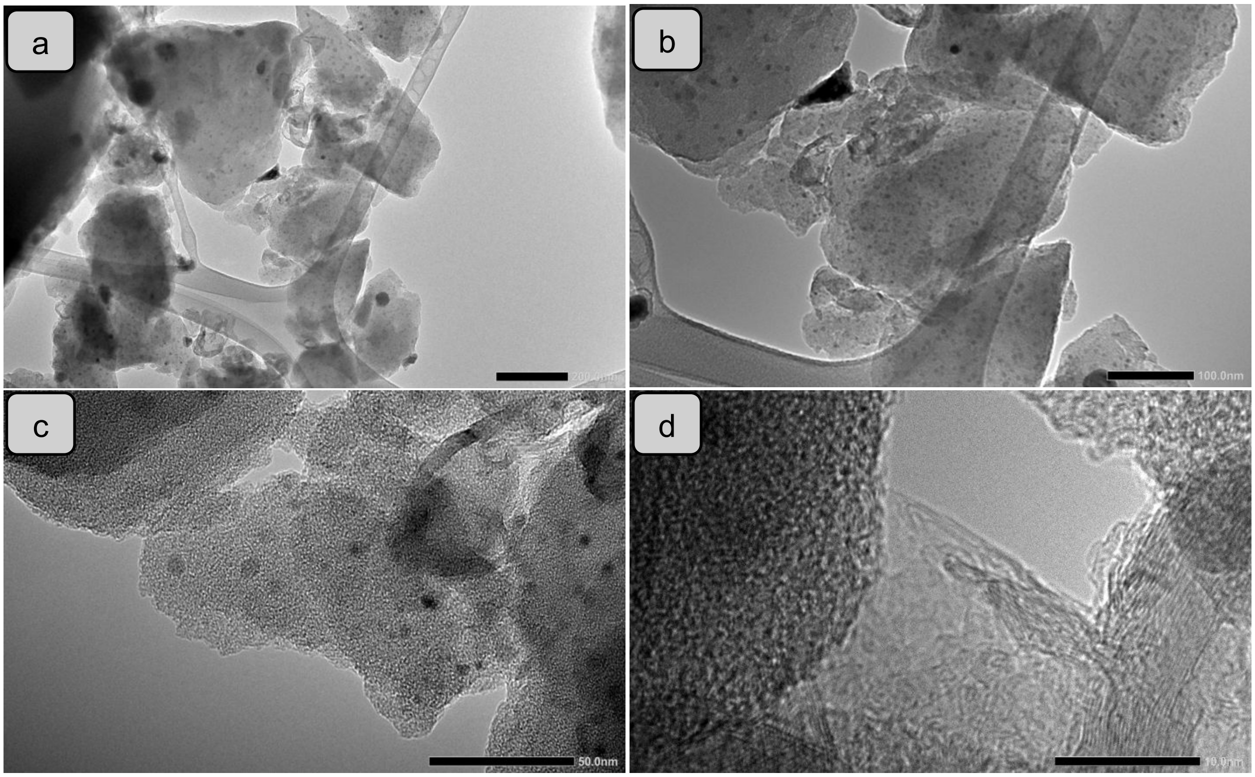

Figure S11: TEM images of BisA-Fu-15wt 700 °C from 200 nm (a) to 10 nm (d).

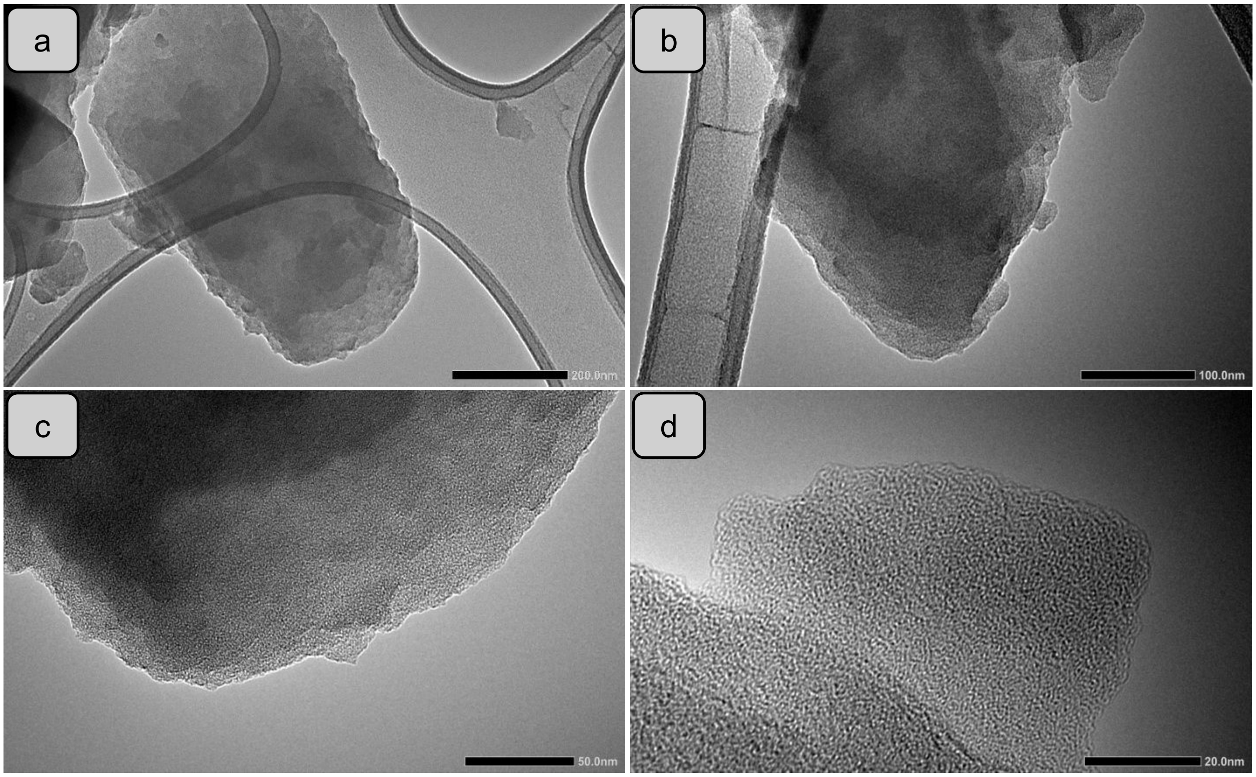

Figure S12: TEM images of BisA-AnFu-Neat 700 °C from 200 nm (a) to 20 nm (d).

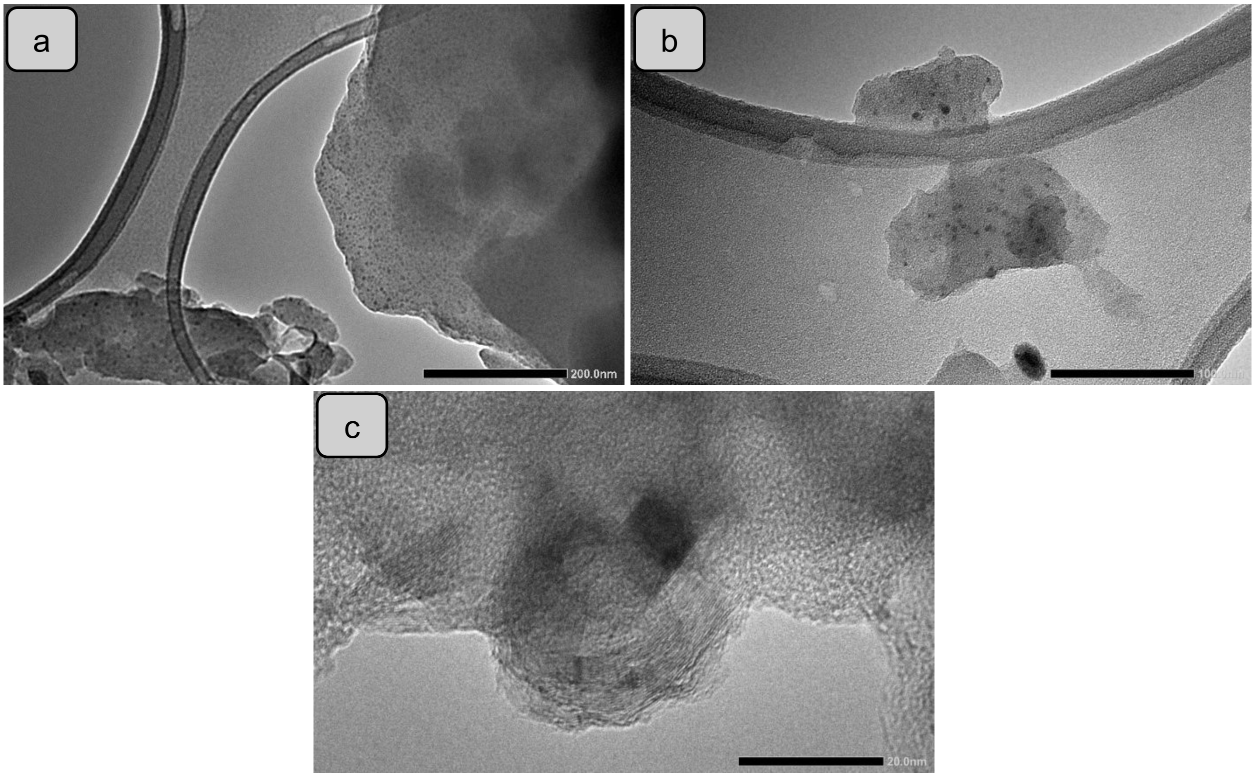

Figure S13: TEM images of BisA-AnFu-15wt 700 °C from 200 nm (a) to 20 nm (c).

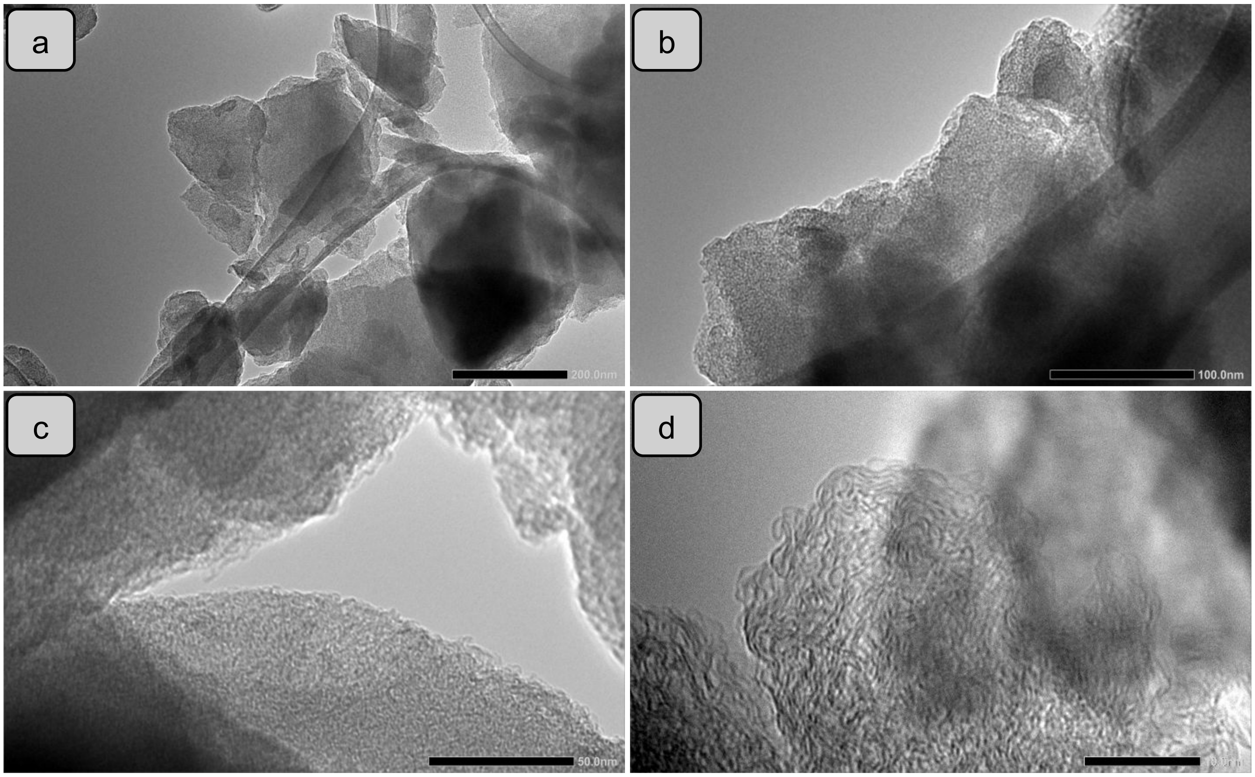

Figure S14: TEM images of BisA-Fu-Neat 1000 °C from 200 nm (a) to 10 nm (d).

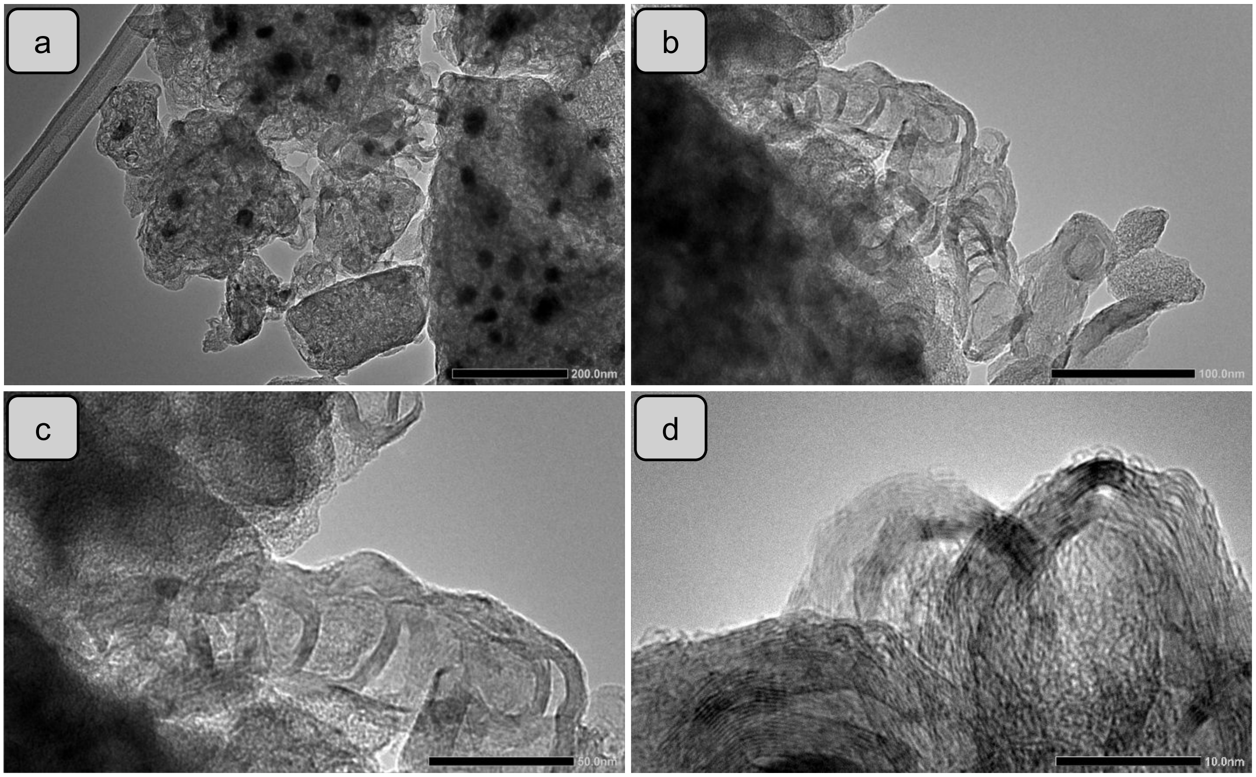

Figure S15: TEM images of BisA-Fu-15wt 1000 °C from 200 nm (a) to 10 nm (d).

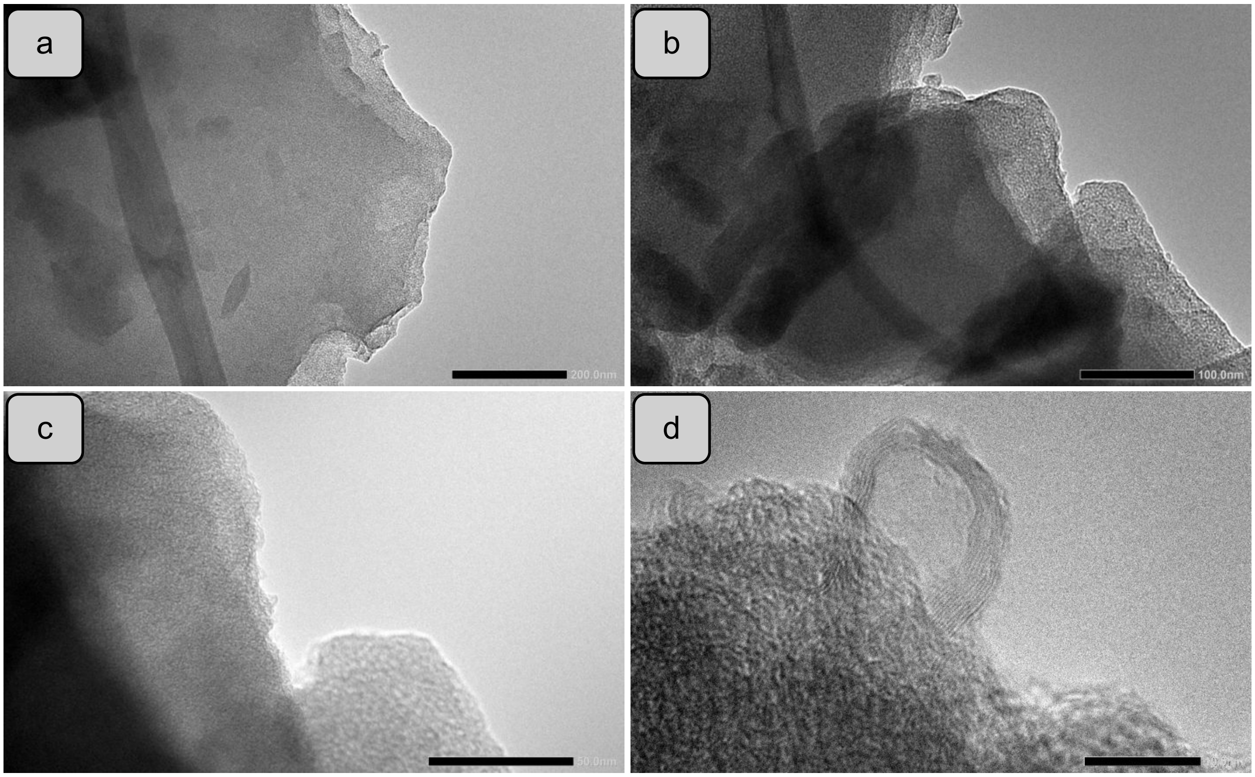

Figure S16: TEM images of BisA-AnFu-Neat 1000 °C from 200 nm (a) to 10 nm (d).

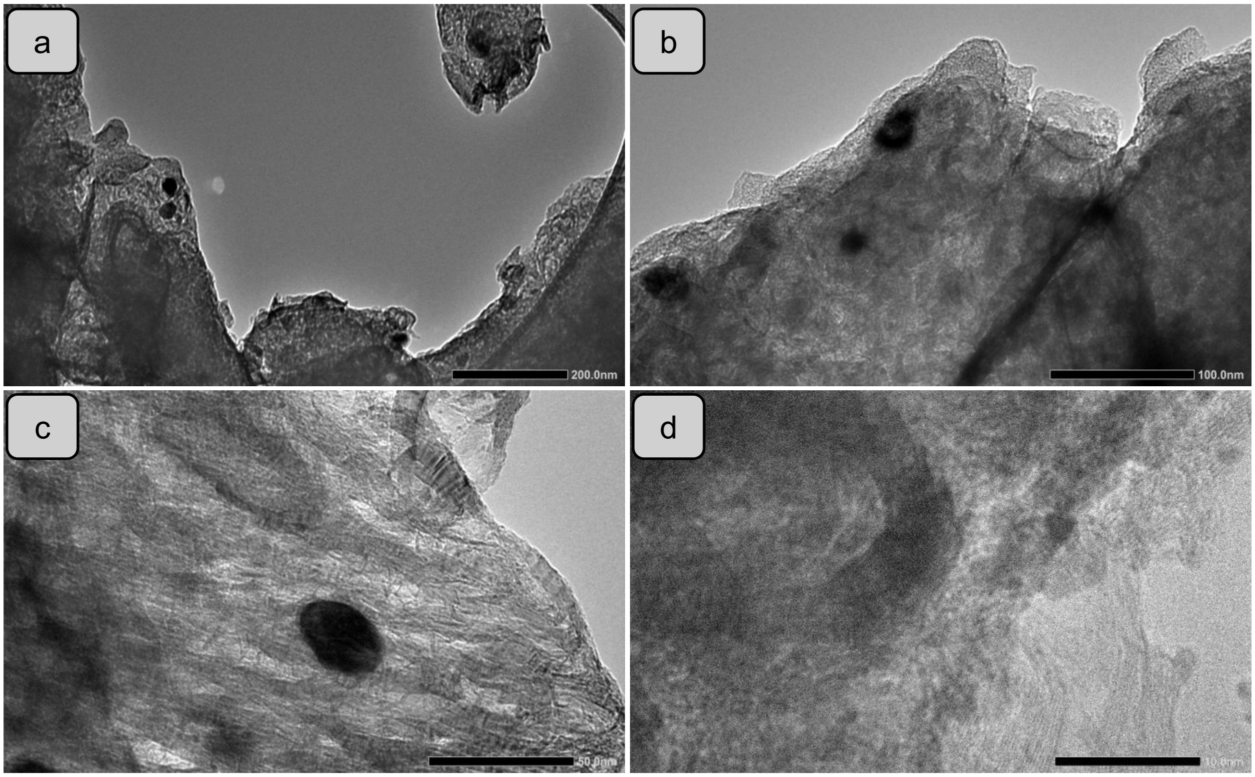

Figure S17: TEM images of BisA-AnFu-15wt 1000 °C from 200 nm (a) to 10 nm (d)
